# VPS13A Deficiency Leads to Impaired Lipid Distribution and Alteration of Mitochondrial Calcium Homeostasis in Fibroblasts of VPS13A Disease Patients

**DOI:** 10.1002/mds.70177

**Published:** 2026-01-19

**Authors:** Dajana Grossmann, Adrian Spranger, Emily Fischer, Johanna W. Schubarth, Jenny Leopold, Hannes Glaß, Sebastian Klicker, My Uyen Dang Thi, Anna Elisabeth Bartalis, Andreas Hermann, Kevin Peikert

**Affiliations:** ^1^ Translational Neurodegeneration Section “Albrecht Kossel”, Department of Neurology, University Medical Center Rostock, University of Rostock Rostock Germany; ^2^ Institute for Medical Physics and Biophysics, Medical Faculty, Leipzig University Leipzig Germany; ^3^ Center for Transdisciplinary Neurosciences Rostock (CTNR), University Medical Center Rostock, University of Rostock Rostock Germany; ^4^ German Center for Neurodegenerative Diseases (DZNE) Rostock/Greifswald Rostock Germany; ^5^ United Neuroscience Campus Lund‐Rostock (UNC) Rostock Germany

**Keywords:** Bridge‐like lipid‐transport proteins (BLTPs), calcium, lipids, MERCS, mitochondria, VPS13A

## Abstract

**Background:**

Membrane contact sites are crucial for the exchange of ions or lipids and thus are critical for the function and maintenance of organelles. VPS13A is a membrane‐residing, bridge‐like protein connecting two membranes to enable bulk lipid transfer. Loss‐of‐function mutations in the *VPS13A* gene cause VPS13A disease. Previous studies showed alterations of lipid transfer and impaired calcium homeostasis.

**Objective:**

Although membrane contact sites are becoming increasingly important in neurodegenerative disease research, their contribution to cellular homeostasis is still unclear. We attempted to investigate the consequences of loss of VPS13A function on membrane contact sites and related mechanisms in the context of VPS13A disease.

**Methods:**

VPS13A‐deficient patient‐derived fibroblasts were compared with fibroblasts from healthy donors. Specific dyes, labeled fatty acids, and a specific marker for mitochondrial–endoplasmic reticulum contact sites were used to investigate lipid transfer and distribution in involved organelles. Mitochondrial calcium handling was investigated using the calcium indicator Rhod‐2, AM. Images were obtained by super‐resolution microscopy using Airyscan2 technology.

**Results:**

We observed a general disturbance of membrane contact sites in VPS13A disease, accompanied by a reduction in lipid droplet formation, diminished lipid transfer into mitochondria, and unusual mitochondrial calcium uptake behavior in VPS13A disease fibroblasts.

**Conclusions:**

Loss of VPS13A causes alterations beyond an impairment of lipid shuttling, which includes a dysregulation of membrane contact sites as well as impaired mitochondrial calcium handling. Accordingly, our findings contribute significantly to the understanding of mechanisms directly or indirectly linked to the function of VPS13A. © 2026 The Author(s). *Movement Disorders* published by Wiley Periodicals LLC on behalf of International Parkinson and Movement Disorder Society. © 2026 The Author(s). *Movement Disorders* published by Wiley Periodicals LLC on behalf of International Parkinson and Movement Disorder Society.

1

VPS13A disease, formerly known as chorea‐acanthocytosis, is an ultra‐rare condition caused by autosomal‐recessive mutations in the *VPS13A* gene, typically causing loss of the protein.^1^, ^2^ VPS13A disease is a neurodegenerative disorder resulting in reduced quality of life and life expectancy,^1^, ^2^ characterized by morphologically altered red blood cells, so called acanthocytes, and neurological manifestations, including epileptic seizures, cognitive and behavioral impairment, and movement disorders such as chorea, dystonia, or parkinsonism arising from the degeneration of the basal ganglia.^1^, ^3^, ^4^

VPS13A is involved in many cellular processes like autophagy, calcium homeostasis, and the function of mitochondria and the endoplasmic reticulum (ER).^5^, ^6^, ^7^, ^8^, ^9^, ^10^ Nevertheless, only recently was VPS13A identified as a member of the bridge‐like lipid transfer protein (BLTP) family.^11^, ^12^ BLTPs reside at membrane contact sites (MCS) between organelles where they facilitate lipid transfer in a non‐vesicular manner.^12^ VPS13A is specifically localized at MCS between the ER and mitochondria, ER and lipid droplets, ER and endosomes, or ER and the plasma membrane. ^1^, ^12^, ^13^, ^14^, ^15^

Mitochondria–ER contact sites (MERCS) orchestrate cellular calcium homeostasis, lipid transfer, and mitochondrial function,^16^, ^17^, ^18^ mechanisms that are commonly impaired in neurodegeneration.^19^, ^20^, ^21^ Depending on their function, MERCS consist of different protein complexes which, contingent on the proteins involved, form different types of MERCS with variable distances between the ER and mitochondrial membranes. Wide appositions function as a hub for mitochondrial quality control^22^ or may facilitate phospholipid transfer (eg, for autophagosome formation).^23^, ^24^ In contrast, narrow MERCS have been ascribed a role in the regulation of calcium homeostasis.^23^, ^25^

Based on current knowledge of VPS13A function, we hypothesized that loss of VPS13A not only affects lipid transfer and distribution, but also directly or indirectly affects different MCS, thus further disturbing lipid and calcium homeostasis. Therefore, we aimed to study MCS, lipid transfer, and calcium exchange at MERCS for the first time in VPS13A‐deficient patient‐derived fibroblasts.

## 2 Materials and Methods

### 2.1 Cell Culture

Primary fibroblasts of unrelated controls and VPS13A disease patients (Table 1) were cultivated in Dulbecco's Modified Eagle Medium (DMEM) (+ 1% penicillin/streptomycin, + 10% fetal bovine serum; GIBCO by Thermo Fisher Scientific: 31966‐021). Mycoplasma Test Kit (Promo Cell: PK‐CA91‐1096) was used to test for mycoplasma.

**TABLE 1:** Overview of fibroblast lines

Cell line	Sex	Clinical feature	VPS13A Western blot	Age at biopsy (years)	Mutation in the *VPS13A* gene
C_1	M	Healthy	Present	34	–
C_2	M	Healthy	Present	34	–
C_3	F	Healthy	Present	48	–
D_1	F	Parkinsonism, dystonia, epilepsy, peripheral neuropathy	Absent	46	c.4115‐2923_4957‐185inv (homozygous)
D_2	M	Chorea, epilepsy, peripheral neuropathy	Absent	28	c.6059delC, c.4115‐2923_4957‐185inv (compound heterozygous)

Abbreviations: M, male; F, female.

The use of patient‐derived fibroblasts was in accordance with the Declaration of Helsinki (World Medical Association, 1964) and approved by the Ethical Committee of the Technische Universität Dresden (EK 393122012 and EK 45022009) and the Universität Rostock (A2019‐0134), Germany. A declaration of consent was received from all participants, including for the publication of any research results.

### 2.2 Immunostainings and LipidTOX Staining

Fibroblasts were fixed in 4% paraformaldehyde (Morphisto: 11762.02500), permeabilized in 0.2% TritonX‐100 (Roth: 3051.3; in phosphate‐buffered saline [PBS]), blocked in Pierce™ protein‐free blocking buffer (Thermo Fisher Scientific: 37572), then incubated with primary antibodies against TOM20 (Santa Cruz: sc‐17764; 1:1000), PMP70 (Invitrogen: PA1‐650; 1:1000), Catalase (Cell Signaling Technology: 12980S; 1:1000), or KDEL (Invitrogen: MA5‐34715; 1:1000) and secondary antibodies Alexa Fluor‐488 goat anti‐mouse‐IgG (Invitrogen: A11029; 1:1000) and Alexa Fluor‐647 goat anti‐rabbit‐IgG (Invitrogen: A21245; 1:1000). Samples were stained with LipidTOX red (Invitrogen, Thermo Fisher Scientific: H34476; 1:200) and mounted in DAPI Fluoromount‐G (Southern Biotechnology: 0100–20). Colocalization of different signals was assessed based on the signal overlap area of individual antibody signals, normalized per nuclei, using Fiji v1.53t.^26^

### 2.3 Transfections

Fibroblasts were transfected with 1 μg pDNA (either SPLICS‐Mt‐ER‐short (Addgene: 164108), SPLICS‐Mt‐ER‐long (Addgene: 164107), or pmRFP‐LC3 (Addgene: 21075) using FuseIT DNA (Beniag: 60601). Experiments were conducted 24 hours post‐transfection.

### 2.4 Live Cell Imaging of Lipid Droplets and Lipid Droplet Formation

Fibroblasts were stained with 100 nM MitoTracker Deep Red FM (Cell Signaling Technology: 8778) and 1 μM Bodipy493/503 (Invitrogen, Thermo Fisher Scientific: D3922) for 30 minutes at 37°C. Images were acquired at baseline and after 1 hour and 2 hours of 500 mM oleic acid (Sigma‐Aldrich: 03008) application.

### 2.5 Live Cell Imaging of BodipyC12 Lipid Shuttling into Mitochondria

Fibroblasts transfected with SPLICS constructs (Addgene: 164108 or 164107) were incubated with 1 μM BodipyC12 (Invitrogen, Thermo Fisher Scientific: D3835) for 16 hours, followed by three times rinsing in medium and a washout phase of 1 hour. Cells were stained with 100 nM MitoTracker Deep Red FM for 30 minutes. For starvation, fibroblasts were rinsed three times in Hanks' Balanced Salt Solution (HBSS) (GIBCO by Thermo Fisher Scientific: 14170088) and then incubated in HBSS for 1 or 2 hours.

### 2.6 Thin Layer Chromatography

For detailed method description see Supplementary Material.

### 2.7 Live Cell Imaging of 18:1 NBD‐PS Lipid Shuttling into Mitochondria

18:1 NBD‐phosphatidylserine (PS; Avanti: 810198C‐1M) and 18:1 1,2‐di‐(9Z‐octadecenoyl)‐*sn*‐glycero‐3‐phosphocholine (DOPC; Avanti: 850375C‐25 mg) were mixed in a proportion of 80:20. The lipids were resuspended in Dulbecco's Phosphate‐Buffered Saline (DPBS) (PAN Biotech: P04‐36500). Fibroblasts were loaded with 0.2 mM 18:1 NBD‐PS/DOPC and 100 nM MitoTracker Deep Red FM for 30 minutes at 37°C, then washed in DMEM+/+ and imaged at baseline conditions. For starvation conditions, fibroblasts were rinsed three times in HBSS and imaged after 1 or 2 hours in HBSS.

### 2.8 Live Cell Imaging of Autophagosome Formation

Fibroblasts transfected with pmRFP‐LC3 (Addgene: 21075) were stained with 100 nM MitoTracker Deep Red FM, 0.2 mM 18:1 NBD‐PS/DOPC, and 1 μM Hoechst for 30 minutes at 37°C. Then, fibroblasts were washed once in DMEM+/+ and imaged. Finally, the cells were rinsed three times in HBSS and imaged after 2 hours in HBSS.

### 2.9 Live Cell Imaging of Mitochondrial Calcium Uptake

Fibroblasts transfected with SPLICS‐short were stained with 100 nM MitoTracker Deep Red FM and 1 μM Rhod‐2, AM (Thermo Fisher Scientific: R1244) for 30 minutes at 37°C. Live cell microscopy was performed for 2 minutes at an interval of 10 seconds under baseline conditions. Cells were then treated with dimethyl sulfoxide (DMSO), 1 μM thapsigargin (Sigma‐Aldrich: T9033‐5MG), or a mixture of 1 μM thapsigargin and 10 μM Ru360 (Sigma‐Aldrich: 557440‐500UG) and imaging was continued for 2 minutes.

### 2.10 Microscopy

For microscopy, 5000 fibroblasts per well were grown in eight well high μ‐Slides (Ibidi: 80806–90). All microscope imaging was performed on an inverted AxioObserver.Z1 LSM900 microscope with a super‐resolution Airyscan 2 module, using a 63× 1.4 NA plan apochromat objective (Zeiss). For live cell imaging, the microscope was equipped with a Pecon incubation system to maintain 5% CO_2_ and 37°C. All microscopy data were analyzed using Fiji v1.53t.^26^

### 2.11 Western Blot for VPS13A

Fibroblasts were lysed in radioimmunoprecipitation assay (RIPA) buffer (50 mM Tris‐HCl, pH 7,4; 150 mM CaCl; 1% Triton X‐100; 0.5% Na‐deoxycholate; 0.1% sodium dodecyl sulfate [SDS]; cOmplete, EDTA‐free protease inhibitor cocktail [Roche: 04693132001]) for 30 minutes on ice. Lysates were mixed with 5× urea buffer (200 mM glycin, 25 mM Tris‐HCl, 4 M urea, 10% SDS, 400 mM DTT, 0.02% bromophenol blue; pH 6.8). SDS‐PAGE (polyacrylamide gel electrophoresis) was performed using a 3%–8% Criterion XT Tris‐Acetate Protein Gel (Bio‐Rad: 3450129). Blotting was performed using the Trans‐Blot Turbo RTA Midi 0.2 μm Nitrocellulose Transfer Kit (Bio‐Rad: 1704271). Membranes were blocked in 5% milk (Carl Roth: T145.3; in TBS), then incubated with antibodies against VPS13A (Invitrogen: PA‐54483; 1:500 in 5% skim milk) and β‐actin (Sigma: A5441; 1:10000 in 5% skim milk) and with secondary antibodies donkey anti‐rabbit‐HRP (Invitrogen: A16035; 1:5000 in TBS‐T) and donkey anti‐mouse‐HRP (Invitrogen: AA16017; 1:5000 in TBS‐T). The signal was revealed using Cytiva Amersham ECL Prime (Fisher Scientific/Life Science Cytiva: 10308449 [RPN2232]) on the Li‐COR Odyssey XF.

### 2.12 Western Blot for LC3B‐I/II or Mitochondrial Calcium Uniporter (MCU)

Fibroblasts were lysed in RIPA buffer. SDS‐PAGE was performed using 12% self‐casted polyacrylamide gels. For protein blotting, the Trans‐Blot Turbo TRA Transfer Kit (Biorad: 1704271) was used. Total protein was stained with Revert 700 Total Protein Stain (LICORbio: 926‐11011). Membranes were blocked in 5% milk (Carl Roth: T145.3; in TBS), then incubated with antibodies against LC3B (Cell Signaling Technology: 83506S; 1:1000 in 5% skim milk) or MCU (Proteintech: 26312‐1‐AP; 1:2000 in 5% skim milk) and with secondary antibodies donkey anti‐rabbit‐HRP (Invitrogen: A16035; 1:5000 in TBS‐T) or donkey anti‐mouse‐HRP (Invitrogen: AA16017; 1:5000 in TBS‐T). The signal was revealed using Cytiva Amersham ECL Prime.

### 2.13 Statistics

GraphPad Prism version 6.07 was used for statistical analyses. Data were first tested for normal distribution by a D'Agostino–Pearson omnibus normality test. Statistical tests were used as indicated in the respective figure legends. All experiments were independently repeated at least three times, defined by separate passages. For analysis, we pooled data from the control fibroblasts from three independent healthy donors and fibroblasts from two VPS13A disease patients. Results of individual lines are depicted in the Supplemental Material.

## 3 Results

### 3.1 Impaired Lipid Droplet Formation in Patient‐Derived Fibroblasts

**FIG. 1:**
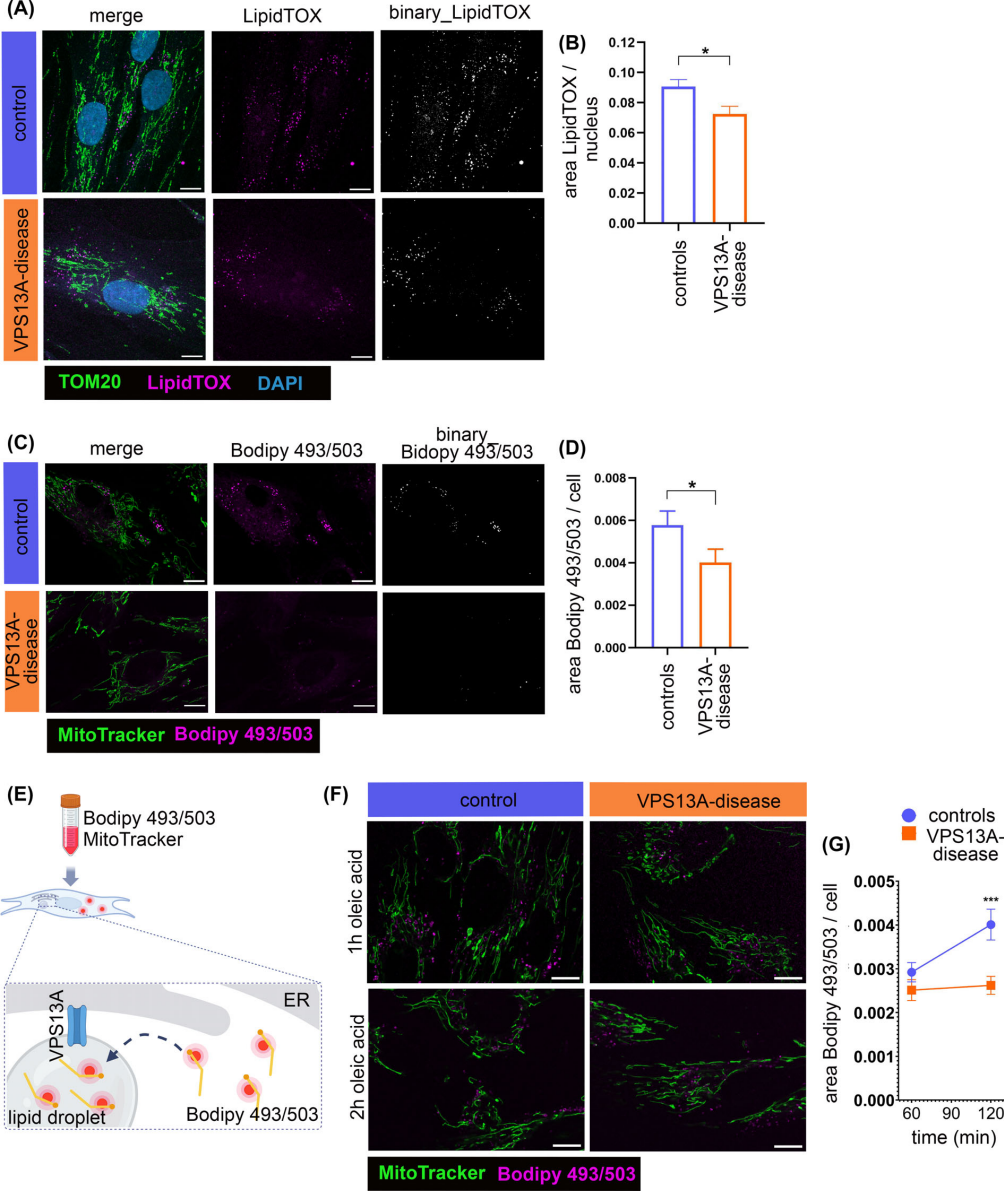
Impaired lipid droplet formation in VPS13A‐deficient fibroblasts. (A) Fixed fibroblasts were immunolabeled with an antibody against TOM20 (green). Lipid droplets were stained with LipidTOX (magenta). The nucleus was stained with DAPI (blue). (B) Quantification of LipidTOX area outside of the TOM20 signal, indicative for lipid droplets, normalized per nucleus. Statistical significance calculated by Mann–Whitney test (controls: *N* = 14; VPS13A disease: *N* = 9). (C) Fibroblasts were stained with MitoTracker Green FM (green) and Bodipy493/503 (magenta). (D) Quantification of the Bodipy493/503 signal area outside the MitoTracker signal, indicating lipid droplets, normalized per cell. Statistical significance calculated by Mann–Whitney test (controls: *N* = 10; VPS13A disease: *N* = 3). (E) Schematic illustration of lipid droplet formation assay using MitoTracker Deep Red FM and Bodipy493/503: Fibroblasts were stained with Bodipy493/503 and MitoTracker Deep Red FM. The Bodipy493/503 was taken up into the cytosol. Oleic acid treatment drives Bodipy493/503 incorporation into newly formed lipid droplets. Image created with Biorender.com. (F) Fibroblasts were stained with MitoTracker Deep Red FM (green) and Bodipy493/503 (magenta) and loaded with oleic acid. Images were acquired after 1 hour and 2 hours. (G) Quantification of the Bodipy493/503 signal after 1 hour and 2 hours of oleic acid incubation, normalized per cell. Statistical significance calculated by two‐way analysis of variance (ANOVA) with Sidak's multiple comparison test (controls: *N* = 8; VPS13A disease: *N* = 6). Scale bars indicate 10 μm. All data are mean ± standard error of the mean (SEM). *P ≤ 0.05, ***P ≤ 0.001. [Color figure can be viewed at wileyonlinelibrary.com]

Primary fibroblasts of three unrelated healthy controls and two unrelated VPS13A disease patients were used (for details see Table 1). Patient‐derived fibroblasts showed absence of VPS13A protein in Western blot analysis (Fig. S1). VPS13A is crucial for lipid droplet formation, but to date has only been reported in non‐patient derived cells.^27^, ^28^ We used LipidTOX, which stains neutral lipids and is widely used to visualize lipid droplets.^27^, ^28^ VPS13A‐deficient fibroblasts showed significantly fewer lipid droplets (Figs. 1A,B and S2A). This observation was further verified by quantification of Bodipy493/503, which also stains neutral lipids (Figs. 1C,D and S2B, ^28^). We next analyzed lipid droplet formation. For this purpose, fibroblasts were incubated with Bodipy493/503 and lipid droplet formation was forced with the mono‐unsaturated fatty acid, oleic acid (Fig. 1E, ^28^). Oleic acid‐induced lipid droplet formation was significantly reduced in VPS13A‐deficient fibroblasts (Figs. 1F,G and S2C). Our findings demonstrate reduced formation of lipid droplets in fibroblasts from patients with VPS13A disease.

### 3.2 Alterations of MCS in VPS13A‐Deficient Fibroblasts

**FIG. 2:**
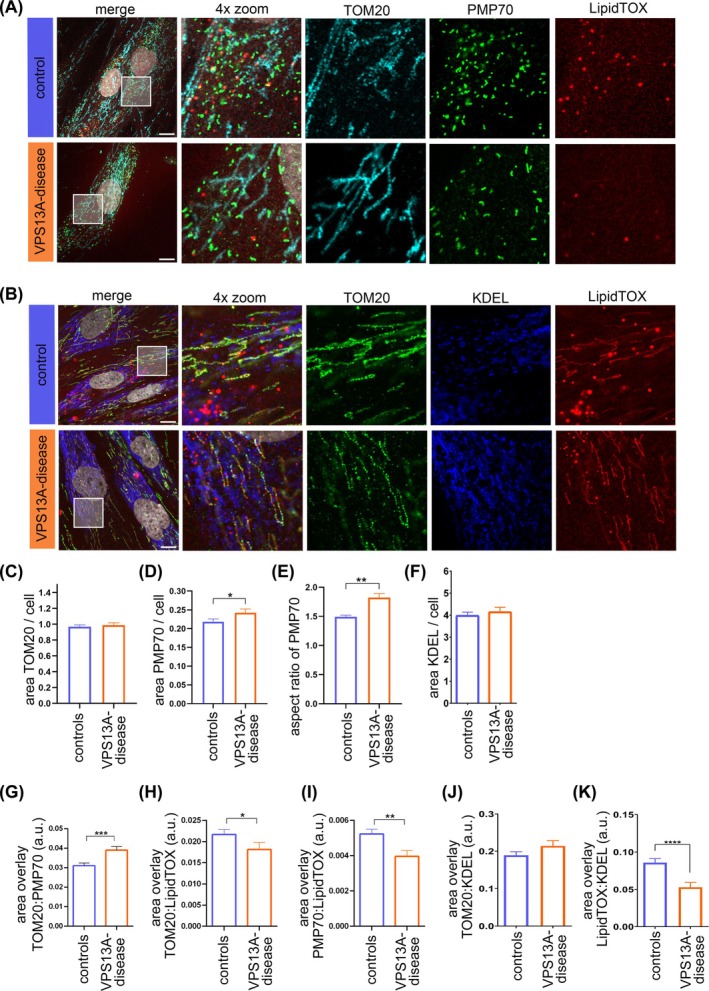
Alterations of membrane contact sites (MCS) in VPS13A‐deficient fibroblasts. (A) Fixed fibroblasts were immunolabeled with an antibody against TOM20 (cyan) and PMP70 (green), then stained with LipidTOX (red) and DAPI (grey). Inlets show areas of 4× magnification. (B) Fixed fibroblasts were immunolabeled with antibodies against TOM20 (green) and KDEL (blue) and stained with LipidTOX (red) and DAPI (grey). Scale bars indicate 10 μm. Quantification of the (C) TOM20 and the (D) PMP70 signal area, normalized per cell (controls: *N* = 14; VPS13A disease: *N* = 9). (E) The aspect ratio of peroxisomes was assessed as the ratio of the minor axis versus the major axis of the PMP70 signal (controls: *N* = 14; VPS13A disease: *N* = 9). (F) Quantification of the KDEL signal area per cell (controls: *N* = 9; VPS13A disease: *N* = 6). Analysis of the overlay area of the signals (G) of TOM20 and PMP70 (controls: *N* = 14; VPS13A disease: *N* = 9), (H) TOM20 and LipidTOX (controls: *N* = 14; VPS13A disease: *N* = 9), (I) PMP70 and LipidTOX (controls: *N* = 14; VPS13A disease: *N* = 9), (J) TOM20 and KDEL (controls: *N* = 9; VPS13A disease: *N* = 6), and (K) KDEL and LipidTOX (controls: *N* = 9; VPS13A disease: *N* = 6); normalized per cell. All data are mean ± standard error of the mean (SEM). Statistical significance calculated by Mann–Whitney test. *P ≤ 0.05, **P ≤ 0.01, ***P ≤ 0.001, ***P ≤ 0.0001. [Color figure can be viewed at wileyonlinelibrary.com]

Since the synthesis of various lipids requires the transfer of lipids between the ER, mitochondria, and peroxisomes, mediated in part by VPS13A, we wanted to investigate the possible phenotypes of these organelles. To this end, we immunolabeled fibroblasts with antibodies against the mitochondrial marker TOM20 and the peroxisome protein PMP70 (Fig. 2A), as peroxisomes together with mitochondria play a central role in lipid metabolism.^29^, ^30^ The ER, a crucial organelle for the synthesis of neutral lipids,^31^ was visualized using an antibody against KDEL (Fig. 2B). Neutral lipids were stained with LipidTOX. While the overall area of TOM20 signal was not different between fibroblast lines (Figs. 2C and S3A), analysis of the PMP70 signal revealed increased peroxisome area (Figs. 2D and S3B). Of note, peroxisomes appear more elongated in VPS13A‐deficient fibroblasts, as indicated by an increased aspect ratio (Figs. 2A,E and S3C). Additionally, we used catalase as another peroxisome marker, demonstrating a slight, yet not significant, increase in peroxisome aspect ratio (Fig. S3F–H). However, the circularity of catalase‐labeled peroxisomes was reduced, pointing to alterations in shape (Fig. S3I,J). Quantification of the KDEL signal area showed no differences (Figs. 2F and S3M). The analysis of overlapping areas of different organelle markers from immunostaining uncovered increased interaction of mitochondria and peroxisomes, indicated by signal overlap of TOM20 and PMP70 (Figs. 2 and S3N), while the interaction of mitochondria (Figs. 2H and S3O) or peroxisomes with lipid droplets (Figs. 2I and S3P) was significantly reduced in VPS13A‐deficient fibroblasts. The overlapping area of TOM20 and KDEL immunostaining was not changed (Figs. 2J and S3Q), while the overlapping area of KDEL with LipidTOX was reduced in VPS13A‐deficient fibroblasts (Figs. 2K and S3R). These results may point to alterations in specific MCS in VPS13A‐deficiency, whereas the reduction in organelle interaction with lipid droplets is likely caused by overall reduction in lipid droplets (Fig. 2).

### 3.3 Impaired Fatty Acid Shuttling in VPS13A‐Deficient Fibroblasts

**FIG. 3:**
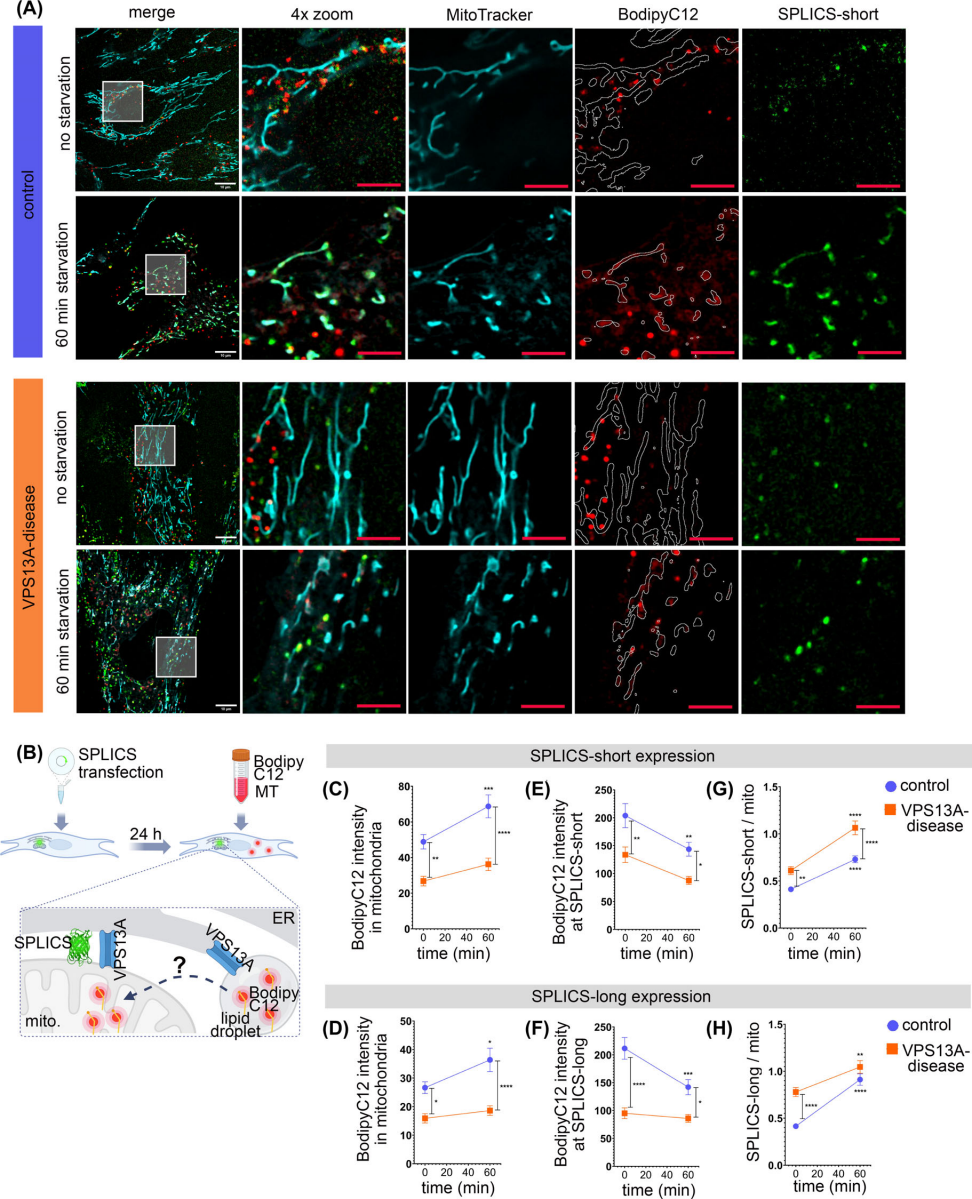
Impaired fatty acid shuttling in VPS13A‐deficient fibroblasts. (A) Fibroblasts were transfected with SPLICS‐short or SPLICS‐long (green), respectively, and 24 hours post‐transfection stained with MitoTracker Deep Red FM (cyan) and BodipyC12 (red). Images were acquired at baseline conditions (0 min), and after 1 hour of starvation. White outlines indicate the outlines of mitochondria within the BodipyC12 image. White scale bar indicates 10 μm, red scale bars indicate 5 μm. (B) Schematic illustration of the experiment setup: fibroblasts expressing SPLICS were stained with BodipyC12 and MitoTracker Deep Red FM (MT). Upon starvation, the BodipyC12 was transferred from the cytosol into the mitochondria. Image generated with Biorender.com. (C),(D) Analysis of mean BodipyC12 intensity inside mitochondria in fibroblasts transfected with (C) SPLICS‐short or (D) SPLICS‐long. (E),(F) Assessment of the mean BodipyC12 intensity at (E) SPLICS‐short or at (F) SPLICS‐long. (G),(H) Quantification of the (G) SPLICS‐short or (H) SPLICS‐long area per mitochondria area. All data are mean ± standard error of the mean (SEM). Statistical significance calculated by two‐way analysis of variance (ANOVA) with Sidak's multiple comparison test (controls: *N* = 9; VPS13A disease: *N* = 6) for SPLICS‐short and (controls: *N* = 6; VPS13A disease: *N* = 4) for SPLICS‐long. *P ≤ 0.05, **P ≤ 0.01, ***P ≤ 0.001, ****P ≤ 0.0001. [Color figure can be viewed at wileyonlinelibrary.com]

**FIG. 4:**
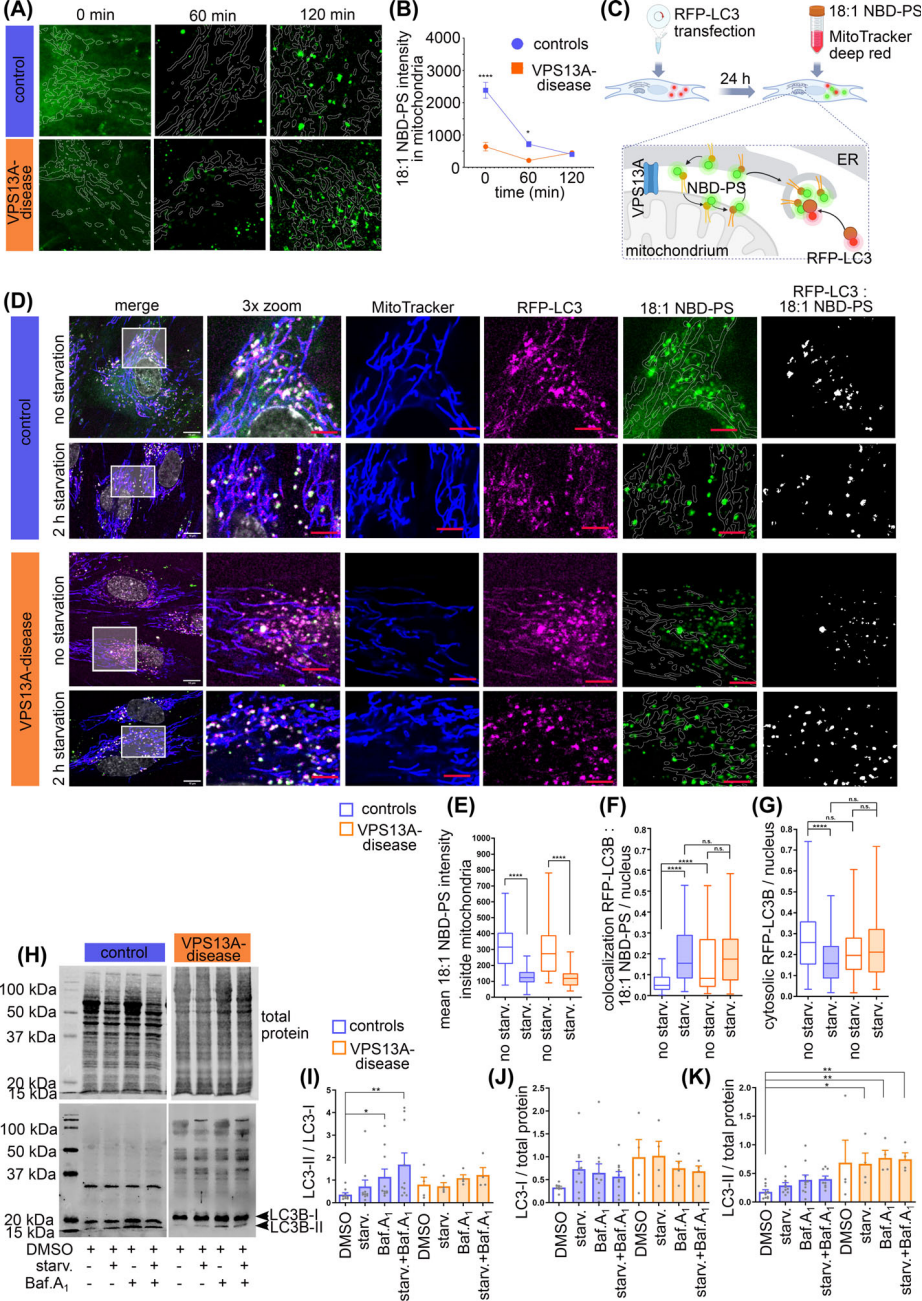
Impaired phospholipid shuttling in VPS13A‐deficient fibroblasts. (A) Fibroblasts were stained with MitoTracker Deep Red FM and loaded with 18:1 NBD‐PS (green). White outlines indicate the mitochondria area inside the 18:1 NBD‐PS image. (B) Quantification of mean 18:1 NBD‐PS signal intensity inside mitochondria (controls: *N* = 9; VPS13A disease: *N* = 8). All data are mean ± standard error of the mean (SEM). Statistical significance calculated by two‐way analysis of variance (ANOVA) with Sidak's multiple comparison test. *P ≤ 0.05, ***P ≤ 0.001. (C) Schematic illustration of the experimental setup: fibroblasts expressing RFP‐LC3B were stained with 18:1 NBD‐PS and MitoTracker Deep Red FM. 18:1 NBD‐PS is integrated into the endoplasmic reticulum (ER) membrane and the mitochondrial membrane. Upon starvation, 18:1 NBD‐PS is converted into phosphatidylethanolamine (PE) in the mitochondria and shuttled to the ER at mitochondria–ER contact sites (MERCS). Labeled PE and RFP‐LC3B are incorporated into the newly formed autophagosome. Image generated with Biorender.com. (D) Fibroblasts were transfected with RFP‐LC3B (magenta) and 24 hours post‐transfection loaded with 18:1 NBD‐PS (green) and MitoTracker Deep Red FM (blue). White scale bars indicate 10 μm, red scale bars indicate 5 μm. (E) Quantification of the mean 18:1 NBD‐PS signal inside the mitochondria. (F) Quantification of colocalization events of the RFP‐LC3B and 18:1 NBD‐PS, indicating autophagosomes. (G) Quantification of the cytosolic RFP‐LC3B signal, outside the mitochondrial as well as outside the 18:1 NBD‐PS signal (controls: *N* = 9; VPS13A disease: *N* = 7). All data are median ± minimum/maximum. Statistical significance calculated by Kruskal–Wallis test with Sidak's multiple comparison test. (H) Image of the Western blot for LC3BI/II protein. Fibroblasts were treated with starvation in Hanks' Balanced Salt Solution (HBSS), or with 100 nM bafilomycinA1, or with a combination of starvation in HBSS and 100 nM bafilomycinA1, for 2 hours. (I) Quantification of the ratio of LC3B‐II to LC3B‐I bands of the Western blot shown in (F). (J) Quantification of LC3B‐I bands, normalized to total protein stain shown in (F). (K) Quantification of LC3B‐II bands, normalized to total protein stain shown in (F). (controls: *N* = 10; VPS13A disease: *N* = 4). Data are mean ± standard error of the mean (SEM). Statistical significance calculated by Kruskal–Wallis test with Dunn's multiple comparison test. *P ≤ 0.05, **P ≤ 0.01, ****P ≤ 0.0001. [Color figure can be viewed at wileyonlinelibrary.com]

Certain lipids are formed from collaboration between the ER and mitochondria at MERCS, requiring lipid transfer by VPS13A and other lipid transfer proteins. The investigation of lipid transfer at MERCS was achieved by transfecting fibroblasts with a split‐GFP contact site sensor (SPLICS) for narrow (SPLICS‐short) or wide MERCS (SPLICS‐long).^23^ The transfection efficiencies between fibroblasts and SPLICS‐short or SPLICS‐long constructs were comparable (Fig. S4G,H). Fibroblasts were subsequently labeled with MitoTracker and the fatty acid BodipyC12 (Fig. 3A). Under starvation, cells switch their metabolism to fatty acid‐fueled oxidative phosphorylation, which can be studied by tracing the transfer of BodipyC12 into mitochondria^32^ (Fig. 3B). The BodipyC12 intensity inside mitochondria was lower in VPS13A‐deficient fibroblasts at baseline. The mitochondrial BodipyC12 intensity increased in controls after 1 hour of starvation, but not in VPS13A‐deficient fibroblasts. This was seen in mitochondria expressing SPLICS‐short (Figs. 3C and S4A) or SPLICS‐long (Figs. 3D and S4B). In controls, the BodipyC12 intensity at SPLICS‐short (Figs. 3E and S4C) or SPLICS‐long (Figs. 3F and S4D) was significantly higher at baseline, compared with VPS13A‐deficient fibroblasts and declined significantly during starvation in controls, but not in VPS13A‐deficient fibroblasts. However, over time the BodipyC12 intensity at SPLICS‐short (Fig. 3E) or SPLICS‐long (Fig. 3F) remained below the BodipyC12 intensity in the controls. Of note, the VPS13A‐deficient fibroblasts displayed elevated amounts of SPLICS‐short and SPLICS‐long at baseline, compared with controls (Figs. 3G,H and S4E,F). Starvation induced an elevation of SPLICS‐short (Fig. 3G) or SPLICS‐long (Fig. 4H) in both control and VPS13A‐deficient fibroblasts. Thin‐layer chromatography (TLC) was performed to evaluate to what extent the artificial fatty acid BodipyC12 was metabolized in our cell model. The results show that a small portion is esterified into phospholipids, while the majority remains unchanged within the time frame of the experiment (Fig. S5). Together, these results suggest less effective lipid uptake into mitochondria in VPS13A‐deficient fibroblasts under starvation conditions. Additionally, in VPS13A‐deficient fibroblasts, the amounts of SPLICS‐short and SPLICS‐long were elevated.

### 3.4 Phospholipid Shuttling and Autophagosome Formation in VPS13A‐Deficient Fibroblasts

For the analysis of phospholipid transfer, mitochondria were loaded with the phospholipid 18:1 NBD‐PS (Fig. 4A). Starvation forces phosphatidylserine (PS) shuttling from ER to mitochondria, where it is converted into phosphatidylethanolamine (PE).^33^, ^34^ Mitochondria in the VPS13A disease fibroblasts took up significantly less 18:1 NBD‐PS. During starvation, the 18:1 NBD‐PS signal in mitochondria decreased in control fibroblasts, until it was indistinguishable from VPS13A‐deficient fibroblasts after 2 hours (Figs. 4B and S6A).

The shuttling of PS between mitochondria and the ER at MERCS is a prerequisite for autophagosome formation.^35^, ^36^ The above‐described assay was combined with the autophagosome marker RFP‐LC3B (Fig. 4C). The overlapping area of 18:1 NBD‐PS with RFP‐LC3B was considered an autophagosome (Fig. 4D). Upon starvation, the 18:1 NBD‐PS signal inside mitochondria decreased (Figs. 4E and S6B), while at the same time the RFP‐LC3B overlapping area with 18:1 NBD‐PS signal increased in the control fibroblasts (Figs. 4F and S6C), indicating the formation of autophagosomes. VPS13A‐deficient fibroblasts showed significantly more autophagosomes at baseline conditions and no further increase upon starvation (Figs. 4F and S6C). Controls showed a reduction in cytosolic RFP‐LC3B upon starvation, while there was no change in the VPS13A‐deficient fibroblasts (Figs. 4G and S6D).

As this assay might be prone to artefacts due to the overexpression of RFP‐LC3B, we analyzed endogenous levels of LC3B by Western blotting (Fig. 4H). The conversion of cytosolic LC3B‐I to membrane‐bound LC3B‐II is indicative of autophagosome formation. In controls, the ratio of LC3B‐II to LC3B‐I was slightly increased by starvation and significantly elevated by bafilomycinA1, suggesting efficient autophagosome formation and turnover via autophagy. In VPS13A‐deficient fibroblasts we did not observe an increase in the LC3B‐II/‐I ratio under any treatment condition (Fig. 4I). The quantification of LC3B‐I protein revealed no significant changes in any conditions (Fig. 4J), while VPS13A‐deficient fibroblasts showed elevated levels of LC3B‐II protein compared with control fibroblasts (Fig. 4K).

Together, these findings suggest that autophagy might be impaired in VPS13A‐deficient fibroblasts.

### 3.5 Alterations of Mitochondrial Calcium Handling in VPS13A‐Deficient Fibroblasts

**FIG. 5:**
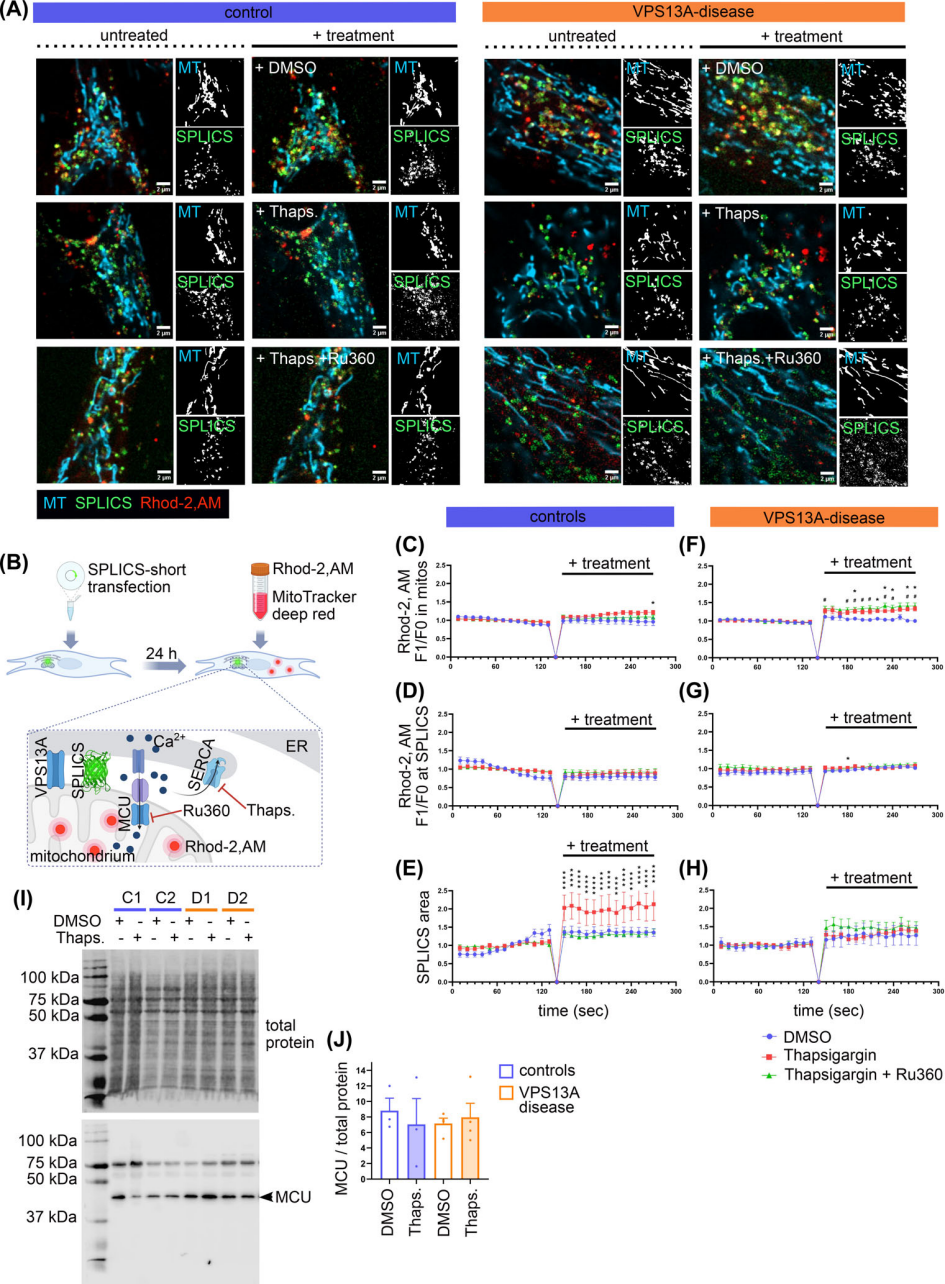
Impaired mitochondrial calcium handling in VPS13A‐deficient fibroblasts. (A) Fibroblasts were transfected with SPLICS‐short (green) and 24 hours later stained with MitoTracker Deep Red FM (cyan) and Rhod‐2, AM (red). Images were acquired for 2 minutes at 10‐second intervals at baseline conditions (left image panels) and for 2 minutes at 10‐seccond intervals after treatment application (dimethyl sulfoxide [DMSO], 1 μM thapsigargin or 1 μM thaspigargin +10 μM Ru360; right image panels). Scale bars indicate 2 μm. (B) Schematic illustration of the experimental setup: fibroblasts expressing SPLICS‐short were stained with Rhod‐2, AM and MitoTracker Deep Red FM. Calcium is shuttled from the endoplasmic reticulum (ER) and the cytosol into the mitochondria at mitochondria–ER contact sites (MERCS), via voltage‐dependent anion channel (VDAC) and mitochondrial calcium uniporter (MCU) calcium channels. Rhod‐2, AM intensity indicates mitochondrial calcium levels, MitoTracker Deep Red FM was used to identify mitochondria. Thapsigargin is an inhibitor of sarcoplasmic/ER calcium ATPase (SERCA), blocking calcium uptake into the ER, resulting in elevation in cytosolic calcium levels. Ru360 inhibits the MCU, preventing mitochondrial calcium uptake. (C) Mean Rhod‐2, AM intensity inside mitochondria in control fibroblasts. (D) Mean Rhod‐2, AM intensity at SPLICS‐short in control fibroblasts. (E) Quantification of SPLICS‐short area in control fibroblasts. (F) Mean Rhod‐2, AM intensity inside mitochondria in VPS13A‐deficient fibroblasts. (G) Mean Rhod‐2, AM intensity at SPLICS‐short in VPS13A‐deficient fibroblasts. (H) Quantification of SPLICS‐short area in VPS13A‐deficient fibroblasts (controls: *N* = 11; VPS13A disease: *N* = 6). All data are mean ± standard error of the mean (SEM). Statistical significance calculated by two‐way analysis of variance (ANOVA) with Dunnett's multiple comparison test. *P ≤ 0.05, **P ≤ 0.01, ****P ≤ 0.0001. (I) Fibroblasts were treated with DMSO or 1 μM thapsigargin for 1 hour and subsequently lyzed for Western blot analysis of MCU protein level. Total protein stain served as loading control. (J) Quantification of the MCU protein bands, normalized to total protein staining. All data are mean ± SEM (controls: *N* = 3; VPS13A disease: *N* = 4). Statistical significance calculated by two‐way ANOVA with Dunnett's multiple comparison test. [Color figure can be viewed at wileyonlinelibrary.com]

Previous studies showed diminished store‐operated calcium entry (SOCE) in VPS13A‐deficient cells.^10^, ^37^, ^38^ Since MERCS are crucially involved in calcium transfer between the ER and mitochondria, we analyzed mitochondrial calcium uptake using a protocol previously published by our group.^39^ Narrow MERCS, which are involved in this process,^23^, ^39^ were visualized by SPLICS‐short, mitochondria were stained with MitoTracker, and calcium was assessed by Rhod‐2, AM (Fig. 5A). Thapsigargin was used to inhibit the sarcoplasmic/ER calcium ATPase (SERCA), preventing calcium uptake by the ER and forcing mitochondria to buffer calcium.^40^ The mitochondrial calcium uptake was blocked by the MCU inhibitor Ru360^41^ (Fig. 5B).

In controls, thapsigargin but not the combination of thapsigargin and Ru360 induced a significant elevation in mitochondrial calcium (Figs. 5C and S7A,D,G), while calcium levels remained unchanged at SPLICS‐short (Figs. 5D and S7B,E,H). Thapsigargin induced an increase in the amount of SPLICS‐short (Figs. 5E and S7C,F,I). In VPS13A fibroblasts, thapsigargin increased mitochondrial calcium, but Ru360 did not prevent mitochondrial calcium uptake (Figs. 5F and S7J,M). Calcium levels remained stable at SPLICS‐short (Figs. 5G and S7K,N). The amount of SPLICS‐short did not significantly change under any treatment (Figs. 5H and S7L,O). Our findings point to alterations in mitochondrial calcium uptake and insufficient adaptation of narrow MERCS in response to calcium stress in VPS13A‐deficient fibroblasts. The MCU is the main channel responsible for mitochondrial calcium influx.^41^, ^42^, ^43^ Western blot analysis showed no differences in MCU expression between control and VPS13A‐deficient fibroblasts (Fig. 5I,J).

## 4 Discussion

Our systematic evaluation revealed that patient‐derived fibroblasts with loss of VPS13A exhibit reduced lipid droplet formation, alterations in various MCS, impaired fatty acid transfer at MERCS, changes in autophagy, and disturbances in mitochondrial calcium uptake.

VPS13A resides at MCS to facilitate the transfer of lipids from one membrane to the other.^12^ We found that contact sites between mitochondria and peroxisomes were increased in VPS13A‐deficient fibroblasts, whereas contact sites between peroxisomes, the ER, or mitochondria with lipid droplets were decreased. The latter was likely caused by an overall reduction in lipid droplets. Quantification of SPLICS suggests an elevation of MERCS in VPS13A‐deficient fibroblasts. There is currently limited literature addressing the phenotypes of MERCS and lipid droplets in cell models with VPS13A deficiency. Our results are in line with the work of Chen and colleagues, showing a reduction in lipid droplets in CRISPR/Cas9 gene edited VPS31A knockout U‐2OS cells,^27^ while another study in VPS13A knockout MRC5 cells showed reduction in SPLICS‐short and SPLICS‐long and an increase in lipid droplets.^28^ This study identified VPS13A at ER–lipid droplet contact sites to facilitate the lipid transfer required for lipid droplet formation.^28^ The contradicting results highlight the need for more research to investigate the underlying mechanisms. Alterations in lipid droplets have been implicated also in other neurodegenerative diseases such as Parkinson's,^44^, ^45^ Huntington's^46^, ^47^ or Alzheimer's disease.^48^ One possibility is that MERCS and lipid droplets are regulated differently in different tissues, depending on the specific requirements of different cell types. Lipid droplets are dynamic organelles, which are fueled with lipids from the ER^49^ and serve as storage organelles to provide fatty acids for β‐oxidation to mitochondria under conditions of high energy demand.^50^, ^51^ Furthermore, it should be emphasized that unlike other studies with an isogenic background, our study was the first to use fibroblasts from unrelated control subjects and VPS13A disease patients with the persons' individual genetic background, which could conceivably contribute to VPS13A‐related pathology. However, this contributes to the high variability in some experiments, which, together with the small number of fibroblast lines, is a limitation of our study and calls for further validation work in the future.

It is currently assumed that VPS13A transfers phospholipids between membranes.^12^, ^52^ We hypothesized that loss of VPS13A may lead to a general disruption of lipid metabolism, and we investigated fatty acid transfer in mitochondria under metabolic stress. Our results indicate a reduction in fatty acid transfer into mitochondria. Yet, this does not indicate that VPS13A directly transfers fatty acids. However, it is noteworthy that for VPS13D, which is structurally and functionally closely related to VPS13A,^53^ a function in fatty acid transfer between lipid droplets and mitochondria has been described.^54^ Our results could, however, be indirectly associated with loss of VPS13A in that the impaired fatty acid uptake could be a sign of a general disturbance of lipid distribution and/or metabolism.

We furthermore showed alterations in autophagy in VPS13A‐deficient fibroblasts. Previous studies described impaired autophagic flux in *VPS13A*‐knockdown HeLa cells^55^ and during erythroid maturation of patients with VPS13A disease.^8^ Impaired autophagy might arise from an endo‐/lysosomal dysfunction, thereby inhibiting autophagic turnover.^56^ VPS13A interacts with the lipid scramblase ATG9A to facilitate phospholipid transfer at MERCS for autophagosome formation.^57^ Of note, the formation of autophagosomes is a redundant process and also functions via the lipid transfer protein ATG2 interacting with ATG9A,^57^ consistent with our observations that autophagosome formation is nonetheless functional in VPS13A‐deficient cells. VPS13A and ATG2 belong to the same family of lipid transfer proteins.^35^, ^52^, ^57^, ^58^, ^59^

Additionally, we investigated the influence of VPS13A deficiency on calcium homeostasis. Diminished SOCE, the influx of calcium across the plasma membrane into the cell, was observed in VPS13A‐deficient fibroblasts and induced pluripotent stem cell (iPSC)‐derived neurons.^5^, ^37^, ^38^ However, data on mitochondrial calcium homeostasis are lacking. Narrow MERCS appear to be primarily responsible for calcium flux into mitochondria,^23^, ^39^ and since we have already seen an altered response in narrow MERCS to metabolic stress, we wanted to find out whether the loss of VPS13A also has an effect on narrow MERCS‐mediated mitochondrial calcium homeostasis. Consistent with our previous study,^39^ healthy cells appropriately increase mitochondrial calcium levels upon thapsigargin treatment, along with a dynamic reorganization of narrow MERCS. Surprisingly, however, mitochondria in VPS13A disease fibroblasts accumulated calcium also under conditions of MCU inhibition. The MCU is the main channel for mitochondrial calcium uptake.^41^, ^42^, ^43^ Changes in the composition of the protein complex of MCU, MICU1, and EMRE can lead to alterations in the sensitivity of MCU against Ru360.^60^, ^61^, ^62^ We did not observe alterations in MCU protein levels in VPS13A‐deficient fibroblasts. The inability to structurally reorganize the initially elevated SPLICS‐labeled narrow MERCS under calcium stress is remarkably similar to a previously described phenotype in Parkinson's models with deficiency in the MERCS regulating proteins Parkin or PINK1.^39^

Taken together, our data suggest that VPS13A‐deficiency leads to disturbances of MCS beyond impaired lipid transfer, including changes in mitochondrial calcium handling, in that the mitochondria still take up calcium though the cells were exposed to the MCU inhibitor Ru360. However, further studies are required to determine the extent to which this alteration of calcium handling affects mitochondrial function and maintenance and is related to the previously described changes in SOCE. Also, the link between impaired lipid and calcium transfer at MERCS in VPS13A‐deficient cells warrants further investigation.

## Author Roles

(1) Research Project: A. Conception, B. Design, C. Execution, D. Data Analysis; (2) Statistical Analysis: A. Design, B. Execution, C. Review and Critique; (3) Manuscript Preparation: A. Writing of the First Draft, B. Editing of the Final Version.

D.G.: 1A, 1B, 1C, 1D, 2A, 2B, 3A, 3B.

A.S.: 1C, 1D, 3B.

E.F.: 1C, 1D, 3B.

J.W.S.: 1C, 3B.

J.L.: 1C, 1D, 3B.

H.G.: 1D, 3B.

S.K.: 1C.

M.U.D.T.: 1C.

A.E.B.: 1C, 3B.

A.H.: 1A, 1B, 2C, 3B.

K.P.: 1A, 1B, 3A, 3B.

## Financial Disclosures (for the Preceding 12 Months)

A.H. has received funding from the European Social Fonds, the Federal Ministry of Education and Research, and the Hermann und Lilly Schilling‐Stiftung für medizinische Forschung im Stifterverband. He has received royalties from Elsevier Press and Kohlhammer. K.P. was supported by Rostock Academy of Science (RAS).

## Supporting information


**Supplementary Figure S1.** *Western blot for VPS13A*. Fibroblasts of control lines C1, C2, and C3 and the patient‐derived lines D1 and D2 were lyzed for subsequent Western blot analysis of VPS13A protein. The membrane was incubated with antibodies against VPS13A and β‐actin.

**Supplementary Figure S2. (A)** Quantification of LipidTOX area outside of the TOM20 signal, indicative for lipid droplets, normalized per nucleus. Data from Figure 1B displayed for each individual fibroblast line (control_1: *N* = 4; control_2: *N* = 5; control_3: *N* = 5; VPS13A_1: *N* = 6; VPS13A_2: *N* = 3; n = 10 images per condition). (**B**) Quantification of the Bodipy493/503 signal area outside of MitoTracker signal, indicating lipid droplets, normalized per cell. Data from Figure 1D displayed for each individual fibroblast line (control_1: *N* = 3; control_2: *N* = 3; control_3: *N* = 3; VPS13A_1: *N* = 3; VPS13A_2: *N* = 3; n = 10 images per condition). (**C**) Quantification of the Bodipy493/503 signal after 1 hour and 2 hours of oleic acid incubation, normalized per cell. Data from Figure 1G displayed for each individual fibroblast line (control_1: *N* = 2; control:2: *N* = 3; control_3: *N* = 3; VPS13A_1: *N* = 4; VPS13A_2: *N* = 2; n = 10 images per condition). All data are mean ± SEM.

**Supplementary Figure S3.** (A) Quantification of the TOM20 signal area, normalized per cell. Data from Figure 2C displayed for each individual fibroblast line (control_1: *N* = 4; control_2: *N* = 5; control_3: *N* = 5; VPS13A_1: *N* = 6; VPS13A_2: *N* = 3; *n* = 10 images per condition). (B) Quantification of the PMP70 signal area, normalized per cell. Data from Figure 2D displayed for each individual fibroblast line (control_1: *N* = 4; control_2: *N* = 5; control_3: *N* = 5; VPS13A_1: *N* = 6; VPS13A_2: *N* = 3; *n* = 10 images per condition). (C) The aspect ratio of peroxisomes was assessed as the ratio of the minor axis versus the major axis of the PMP70 signal. Data from Figure 2E displayed for each individual fibroblast line (control_1: *N* = 4; control_2: *N* = 5; control_3: *N* = 5; VPS13A_1: *N* = 6; VPS13A_2: *N* = 3; *n* = 10 images per condition). (D) Circularity of the PMP70 signal (control_1: *N* = 4; control_2: *N* = 5; control_3: *N* = 5; VPS13A_1: *N* = 6; VPS13A_2: *N* = 3; *n* = 10 images per condition). (E) Data from Figure S3D displayed for each individual fibroblast line (controls: *N* = 14; VPS13A disease: *N* = 9; *n* = 10 images per condition). (F) Fibroblasts were fixed and immunostained with an antibody against catalase (red) (Abcam: ab209211; 1:1000) and stained with DAPI (blue). Scale bars indicate 10 μm. (**G**) The aspect ratio of peroxisomes was assessed as the ratio of the minor axis versus the major axis of the catalase signal (controls: *N* = 9; VPS13A disease: *N* = 6). (H) Data from Figure S3G displayed for each individual fibroblast line (control_1: *N* = 3; control_2: *N* = 3; control_3: *N* = 3; VPS13A_1: *N* = 3; VPS13A_2: *N* = 3; *n* = 10 images per condition). (I) Circularity of the catalase signal (controls: *N* = 9; VPS13A disease: *N* = 6). Statistical significance calculated by Mann–Whitney test. **P ≤ 0.01. (J) Data from Figure S3I displayed for each individual fibroblast line (control_1: *N* = 3; control_2: *N* = 3; control_3: *N* = 3; VPS13A_1: *N* = 3; VPS13A_2: *N* = 3; *n* = 10 images per condition). (K) Quantification of the area of the Catalase signal, normalized per cell (controls: *N* = 9; VPS13A disease: *N* = 6). (L) Data from Figure S3K displayed for each individual fibroblast line (control_1: *N* = 3; control_2: *N* = 3; control_3: *N* = 3; VPS13A_1: *N* = 3; VPS13A_2: *N* = 3; *n* = 10 images per condition). (M) Quantification of the KDEL signal area per cell. Data from Figure 2F displayed for each individual fibroblast line (control_1: *N* = 3; control_2: *N* = 3; control_3: *N* = 3; VPS13A_1: *N* = 3; VPS13A_2: *N* = 3; *n* = 10 images per condition). (N) Analysis of the overlay area of the signals of TOM20 and PMP70, normalized per cell. Data from Figure 2G displayed for each individual fibroblast line (control_1: *N* = 4; control_2: *N* = 5; control_3: *N* = 5; VPS13A_1: *N* = 6; VPS13A_2: *N* = 3; *n* = 10 images per condition). **O**) Analysis of the overlay area of the signals of TOM20 and LipidTOX, normalized per cell. Data from Figure 2H displayed for each individual fibroblast line (control_1: *N* = 4; control_2: *N* = 5; control_3: *N* = 5; VPS13A_1: *N* = 6; VPS13A_2: *N* = 3; *n* = 10 images per condition). (P) Analysis of the overlay area of the signals of PMP70 and LipidTOX, normalized per cell. Data from Figure 2I displayed for each individual fibroblast line (control_1: *N* = 4; control_2: *N* = 5; control_3: *N* = 5; VPS13A_1: *N* = 6; VPS13A_2: *N* = 3; *n* = 10 images per condition). (Q) Analysis of the overlay area of the signals of TOM20 and KDEL, normalized per cell. Data from Figure 2J displayed for each individual fibroblast line (control_1: *N* = 3; control_2: *N* = 3; control_3: *N* = 3; VPS13A_1: *N* = 3; VPS13A_2: *N* = 3; *n* = 10 images per condition). (**R**) Analysis of the overlay area of the signals of KDEL and LipidTOX, normalized per cell. Data from Figure 2K displayed for each individual fibroblast line (control_1: *N* = 3; control_2: *N* = 3; control_3: *N* = 3; VPS13A_1: *N* = 3; VPS13A_2: *N* = 3; *n* = 10 images per condition). All data are mean ± SEM.

**Supplementary Figure S4.** (A) Analysis of mean BodipyC12 intensity inside mitochondria in fibroblasts transfected with SPLICS‐short. Data from Figure 3C displayed for each individual fibroblast line (control_1: *N* = 3; control_2: *N* = 3; control_3: *N* = 3; VPS13A_1: *N* = 3; VPS13A_2: *N* = 3; *n* = 10 images per condition). (B) Analysis of mean BodipyC12 intensity inside mitochondria in fibroblasts transfected with SPLICS‐long. Data from Figure 3D displayed for each individual fibroblast line (control_1: *N* = 2; control_2: *N* = 2; control_3: *N* = 2; VPS13A_1: *N* = 2; VPS13A_2: *N* = 2; *n* = 10 images per condition). (C) Analysis of mean BodipyC12 intensity at SPLICS‐short in fibroblasts transfected with SPLICS‐short. Data from Figure 3E displayed for each individual fibroblast line (control_1: *N* = 3; control_2: *N* = 3; control_3: *N* = 3; VPS13A_1: *N* = 3; VPS13A_2: *N* = 3; *n* = 10 images per condition). (D) Analysis of mean BodipyC12 intensity at SPLICS‐long in fibroblasts transfected with SPLICS‐long. Data from Figure 3F displayed for each individual fibroblast line (control_1: *N* = 2; control_2: *N* = 2; control_3: *N* = 2; VPS13A_1: *N* = 2; VPS13A_2: *N* = 2; *n* = 10 images per condition). (E) Quantification of the SPLICS‐short area, normalized per mitochondria area in fibroblasts transfected with SPLICS‐short. Data from Figure 3G displayed for each individual fibroblast line (control_1: *N* = 3; control_2: *N* = 3; control_3: *N* = 3; VPS13A_1: *N* = 3; VPS13A_2: *N* = 3; *n* = 10 images per condition). (F) Quantification of the SPLICS‐long area, normalized per mitochondria area in fibroblasts transfected with SPLICS‐long. Data from Figure 3H displayed for each individual fibroblast line (control_1: *N* = 2; control_2: *N* = 2; control_3: *N* = 2; VPS13A_1: *N* = 2; VPS13A_2: *N* = 2; *n* = 10 images per condition). All data are mean ± SEM. (G) Fibroblasts were transfected with SPLICS‐short or SPLICS‐long (green), respectively, and 24 hours post‐transfection stained with MitoTracker Deep Red FM (red) and Hoechst (blue). Images were obtained on an inverted AxioObserver.Z1 LSM900 microscope, using a 20× 1.4 NA plan apochromat objective (Zeiss). Nuclei were counted manually, based on Hoechst staining. Positively transfected fibroblasts were identified using Fiji v1.53t. (H) The transfection efficiency was quantified based on fibroblasts with positive SPLICS signal per counted nuclei, per image (control_1: *N* = 3; control_2: *N* = 3; control_3: *N* = 3; VPS13A_1: *N* = 3; VPS13A_2: *N* = 3; *n* = 10 images per condition). All data are mean ± SEM.

**Supplementary Figure S5.** Primary fibroblasts were investigated using high performance thin‐layer chromatography (HPTLC). Fibroblasts from three healthy controls *(termed “control_1”, “control_2” and “control_3”)* and two patients with VPS13A disease (“VPS13A_1” and “VPS13A_2”) were cultured as previously described in the Materials and Methods section. For each cell line, three conditions were tested: “w/o” (without starvation/without BodipyC12 (BC12) staining); “BC12” (stained with BodipyC12); and “BC12+HBSS” (stained with BodipyC12 and starved in HBSS). Fibroblasts were cultured in triplicate (three independent passages: *N* = 3) for all conditions and all fibroblast lines. Lipid extraction from cell pellets was performed according to the Folch method^63^, with slight modifications. After the addition of 50 μL of ice‐cold methanol (MS grade, Biosolve), containing 0.1% butylated hydroxytoluene (BHT, Sigma‐Aldrich), to the cell pellet, the solution was transferred to a new Eppendorf tube equipped with a glass insert (Knauer). The original tube was then washed with additional 50 μL of methanol, which was also transferred into the glass insert (total methanol volume: 100 μL). After the addition of 200 μL of ice‐cold chloroform (LiChrosolv®, Supelco), containing 0.1% butylated hydroxytoluene, the samples were shaken for 60 minutes at 600 rpm and 4°C using a mixing block (MB‐102, Biostep). Phase separation was achieved by adding 200 μL of water (MS grade, Biosolve) and further incubation at 600 rpm and 4°C for 10 minutes. The samples were then centrifuged at 10,000 rpm and 4°C for 10 minutes, after which the lower (organic) phase was transferred to a new Eppendorf tube containing a glass insert. The aqueous phase was then re‐extracted by adding 100 μL of chloroform to improve the extraction yield. The samples were shaken for 10 minutes at 600 rpm and 4°C, then centrifuged for 10 minutes at 10,000 rpm at 4°C. The two organic phases were then combined and dried under vacuum using a vacuum centrifuge (RVC 2‐25 CDplus, Christ).
Prior to HPTLC, dried samples were dissolved in 100 μL chloroform. The following lipid standards (all from Avanti Research, Alabaster) were used for the method development: 1‐palmitoyl‐2‐hydroxy‐*sn*‐glycero‐3‐phosphocholine (LPC); N‐palmitoyl‐D‐erythro‐sphingosylphosphorylcholine (SM); 1‐palmitoyl‐2‐oleoyl‐*sn*‐glycero‐3‐phosphocholine (POPC); 1‐palmitoyl‐2‐oleoyl‐*sn*‐glycero‐3‐phospho‐L‐serine (POPS); 1‐palmitoyl‐2‐oleoyl‐*sn*‐glycero‐3‐phosphate (POPA), 1‐palmitoyl‐2‐oleoyl‐*sn*‐glycero‐3‐phosphoethanolamine (POPE) and 1‐palmitoyl‐2‐oleoyl‐*sn*‐glycero‐3‐phospho‐(1'‐rac‐glycerol) (POPG). Additionally, BodipyC12 (BC12) was used as a standard both before and after the lipid extraction protocol, as well as for the extracted cell medium and one cell sample without any treatment. The samples were separated on normal phase (silica gel glass plates, 20 × 10 cm, Merck Millipore) HPTLC plates. For the sample application, a Linomat 5 (CAMAG) combined with a standard Hamilton syringe was used. 1 μL of standard and 5 μL of the cell extracts were applied onto the HPTLC plate. The plate was then developed in a commercially available TLC chamber (CAMAG) using a solvent system of highly purified chloroform/ethanol/water/trimethylamine (30/35/7/35, v/v/v/v).^64^ Pictures of the BodipyC12 dye were acquired using a FLA5000 digital visualizer (FUJIFILM) at 532 nm (Cy3 filter) immediately after HPTLC development. The same HPTLC plate was then stained with primuline (Direct Yellow 59, c = 200 mg/L in acetone/water (8/2, v/v), Sigma‐Aldrich) to allow the visualization of the lipid species using a video densitometric device (Biostep GmbH) by irradiation with UV light (366 nm). Despite the absence of BodipyC12 in the buffer or the tested cell sample, a clear spot with the same retardation factor (RF) as BodipyC12 was observed. This spot belongs to the phenol red of the cell culture medium, which has an excitation wavelength of 552 nm. Consequently, background signals are present within the cell sample, originating from the phenol red of the medium.
BodipyC12 (A, C, E) and lipid (B, D, F) detection was performed following excitation at 532 (BodipyC12) and 366 nm (primuline), respectively. The results shown are from extracted cell culture samples taken from three healthy controls (blue bar) and two patients with VPS13A disease (orange bar). For each cell line, three conditions were tested: without treatment/without BodipyC12 staining (w/o); stained with BodipyC12 (BC12); and stained with BodipyC12 and starved with HBSS (BC12+HBSS). All samples were cultured in three independent biological replicates (*N* = 3). The evaluation of the intensity at 532 nm indicates that the majority of BodipyC12 remains bound to the free fatty acid and there is only minor re‐esterification into phospholipids or apolar lipids. The most prominent spots were assigned to phosphatidylcholine (PC), which is the most abundant phospholipid class in mammalian cells. However, several spots at 532 nm (particularly after HBSS treatment) could not be assigned so far. These unassigned signals are most likely derived from BodipyC12‐containing polar molecules since these spots were absent at 366 nm which is characteristic of lipids.

**Supplementary Figure S6.** (A) Quantification of mean 18:1 NBD‐PS signal intensity inside mitochondria. Data from Figure 4B displayed for each individual fibroblast line (control_1: *N* = 3; control_2: *N* = 3; control_3: *N* = 3; VPS13A_1: *N* = 4; VPS13A_2: *N* = 4; *n* = 10 images per condition). All data are mean ± SEM. (B) Quantification of the mean 18:1 NBD‐PS signal inside the mitochondria. Data from Figure 4E displayed for each individual fibroblast line. (C) Quantification of colocalization events of the RFP‐LC3B and the 18:1 NBD‐PS signal, indicating autophagosomes. Data from Figure 4F displayed for each individual fibroblast line. (D) Quantification of the cytosolic RFP‐LC3B signal, outside the mitochondrial as well as outside the 18:1 NBD‐PS signal. Data from Figure 4G displayed for each individual fibroblast line (control_1: *N* = 3; control_2: *N* = 3; control_3: *N* = 3; VPS13A_1: *N* = 3; VPS13A_2: *N* = 4; *n* = 10 images per condition). All data are median ± minimum/maximum.

**Supplementary Figure S7.** (A) Mean Rhod‐2, AM intensity inside mitochondria in control_1 fibroblasts (*N* = 3). (B) Mean Rhod‐2, AM intensity at SPLICS‐short in control_1 fibroblasts (*N* = 3). (C) Quantification of SPLICS‐short area in control_1 fibroblasts (*N* = 3). (D) Mean Rhod‐2, AM intensity inside mitochondria in control_2 fibroblasts (*N* = 4). (E) Mean Rhod‐2, AM intensity at SPLICS‐short in control_2 fibroblasts (*N* = 4). (F) Quantification of SPLICS‐short area in control_2 fibroblasts (*N* = 4). (G) Mean Rhod‐2, AM intensity inside mitochondria in control_3 fibroblasts (*N* = 4). (H) Mean Rhod‐2, AM intensity at SPLICS‐short in control_3 fibroblasts (*N* = 4). (I) Quantification of SPLICS‐short area in control_3 fibroblasts (*N* = 4). (J) Mean Rhod‐2, AM intensity inside mitochondria in VPS13A_1 fibroblasts (*N* = 3). (K) Mean Rhod‐2, AM intensity at SPLICS‐short in VPS13A _1 fibroblasts (*N* = 3). (L) Quantification of SPLICS‐short area in VPS13A _1 fibroblasts (*N* = 3). (M) Mean Rhod‐2, AM intensity inside mitochondria in VPS13A_2 fibroblasts (*N* = 3). (N) Mean Rhod‐2, AM intensity at SPLICS‐short in VPS13A _2 fibroblasts (*N* = 3). (O) Quantification of SPLICS‐short area in VPS13A _2 fibroblasts (*N* = 3). All data are mean ± SEM.

## Data Availability

The data that support the findings of this study are available from the corresponding author upon reasonable request.
