# Supplementary figures and images for: VPS13A Deficiency Leads to Impaired Lipid Distribution and Alteration of Mitochondrial Calcium Homeostasis in Fibroblasts of VPS13A Disease Patients
Source: Mov Disord. 2026 Jan 19;41(4):856–69. doi: 10.1002/mds.70177 (PMC13067328; doi:10.1002/mds.70177)

Supplementary Figure S1: Western blot for VPS13A

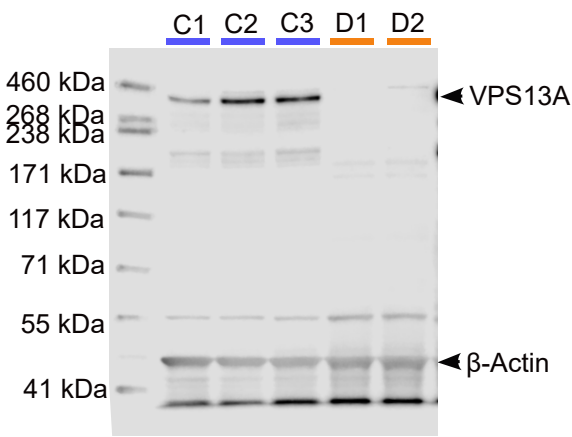

Supplement: Supplementary file 1 — Supplementary Figure S1. Western blot for VPS13A. Fibroblasts of control lines C1, C2, and C3 and the patient‐derived lines D1 and D2 were lyzed for subsequent Western blot analysis of VPS13A protein. The membrane was incubated with antibodies against VPS13A and β‐actin. [file MDS-41-856-s003.pdf]

Figure S2

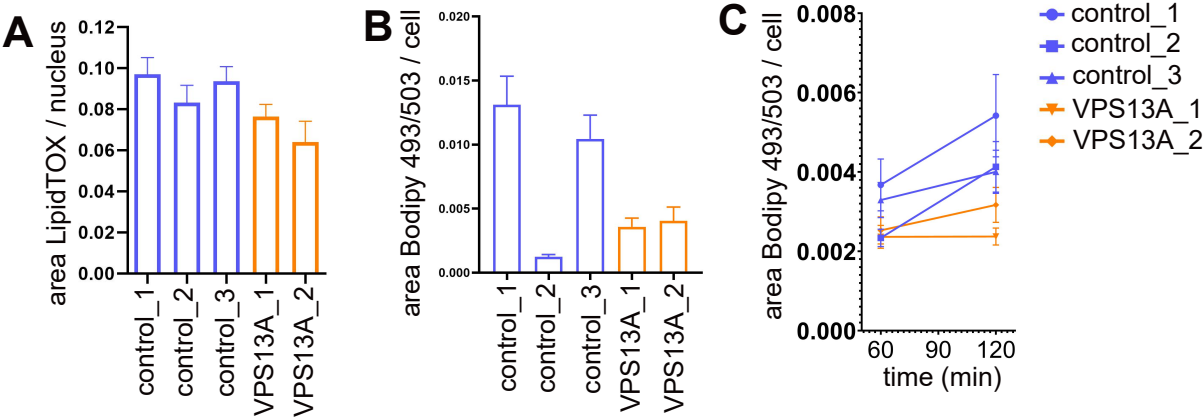

Supplement: Supplementary file 2 — Supplementary Figure S2. (A) Quantification of LipidTOX area outside of the TOM20 signal, indicative for lipid droplets, normalized per nucleus. Data from Figure 1B displayed for each individual fibroblast line (control_1: N = 4; control_2: N = 5; control_3: N = 5; VPS13A_1: N = 6; VPS13A_2: N = 3; n = 10 images per condition). (B) Quantification of the Bodipy493/503 signal area outside of MitoTracker signal, indicating lipid droplets, normalized per cell. Data from Figure 1D displayed for each individual fibroblast line (control_1: N = 3; control_2: N = 3; control_3: N = 3; VPS13A_1: N = 3; VPS13A_2: N = 3; n = 10 images per condition). (C) Quantification of the Bodipy493/503 signal after 1 hour and 2 hours of oleic acid incubation, normalized per cell. Data from Figure 1G displayed for each individual fibroblast line (control_1: N = 2; control:2: N = 3; control_3: N = 3; VPS13A_1: N = 4; VPS13A_2: N = 2; n = 10 images per condition). All data are mean ± SEM. [file MDS-41-856-s004.pdf]

**Figure S3**

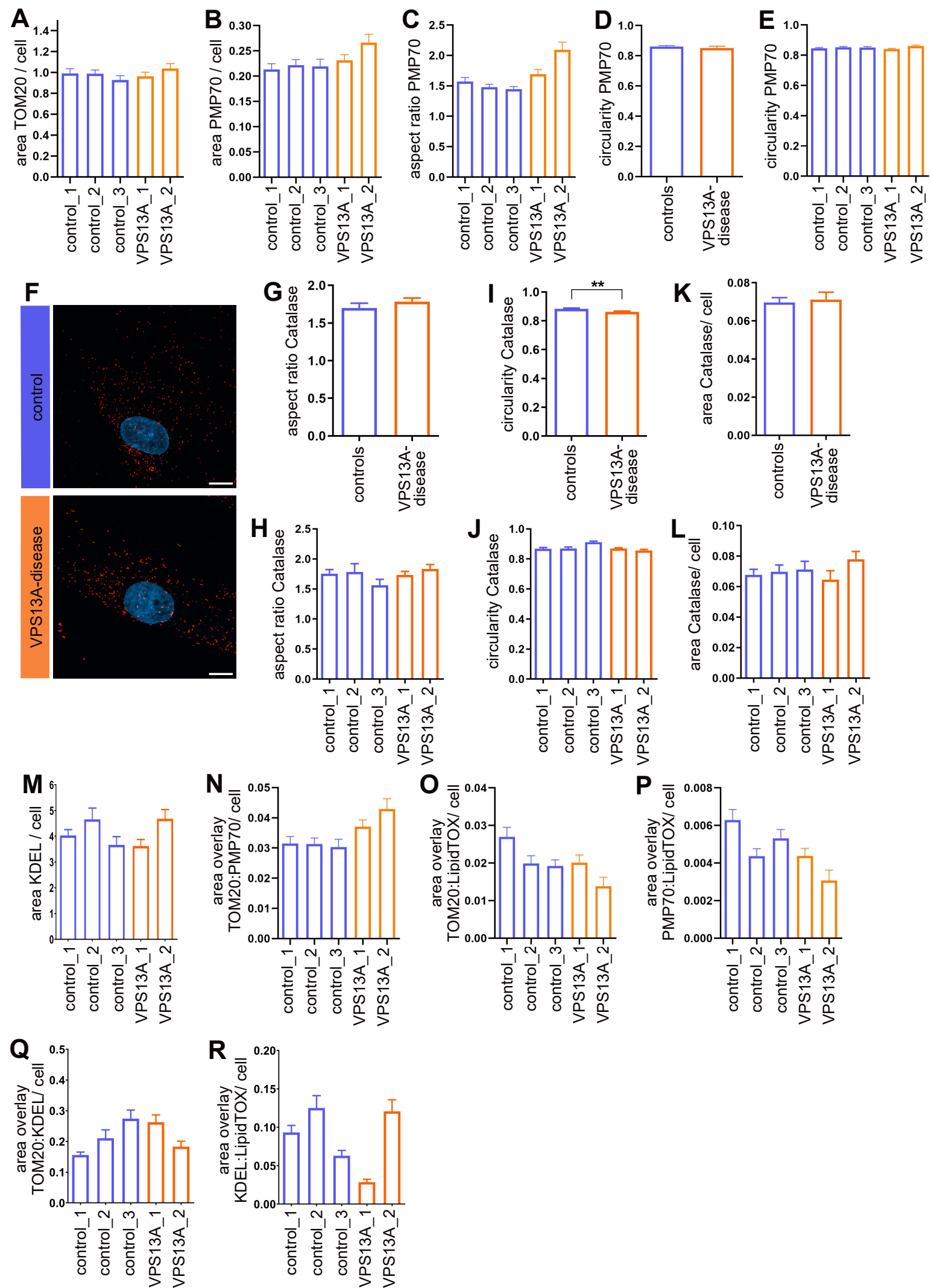

Supplement: Supplementary file 3 — Supplementary Figure S3. (A) Quantification of the TOM20 signal area, normalized per cell. Data from Figure 2C displayed for each individual fibroblast line (control_1: N = 4; control_2: N = 5; control_3: N = 5; VPS13A_1: N = 6; VPS13A_2: N = 3; n = 10 images per condition). (B) Quantification of the PMP70 signal area, normalized per cell. Data from Figure 2D displayed for each individual fibroblast line (control_1: N = 4; control_2: N = 5; control_3: N = 5; VPS13A_1: N = 6; VPS13A_2: N = 3; n = 10 images per condition). (C) The aspect ratio of peroxisomes was assessed as the ratio of the minor axis versus the major axis of the PMP70 signal. Data from Figure 2E displayed for each individual fibroblast line (control_1: N = 4; control_2: N = 5; control_3: N = 5; VPS13A_1: N = 6; VPS13A_2: N = 3; n = 10 images per condition). (D) Circularity of the PMP70 signal (control_1: N = 4; control_2: N = 5; control_3: N = 5; VPS13A_1: N = 6; VPS13A_2: N = 3; n = 10 images per condition). (E) Data from Figure S3D displayed for each individual fibroblast line (controls: N = 14; VPS13A disease: N = 9; n = 10 images per condition). (F) Fibroblasts were fixed and immunostained with an antibody against catalase (red) (Abcam: ab209211; 1:1000) and stained with DAPI (blue). Scale bars indicate 10 μm. (G) The aspect ratio of peroxisomes was assessed as the ratio of the minor axis versus the major axis of the catalase signal (controls: N = 9; VPS13A disease: N = 6). (H) Data from Figure S3G displayed for each individual fibroblast line (control_1: N = 3; control_2: N = 3; control_3: N = 3; VPS13A_1: N = 3; VPS13A_2: N = 3; n = 10 images per condition). (I) Circularity of the catalase signal (controls: N = 9; VPS13A disease: N = 6). Statistical significance calculated by Mann–Whitney test. **P ≤ 0.01. (J) Data from Figure S3I displayed for each individual fibroblast line (control_1: N = 3; control_2: N = 3; control_3: N = 3; VPS13A_1: N = 3; VPS13A_2: N = 3; n = 10 images per condition). ( [file MDS-41-856-s007.pdf]

Figure S4

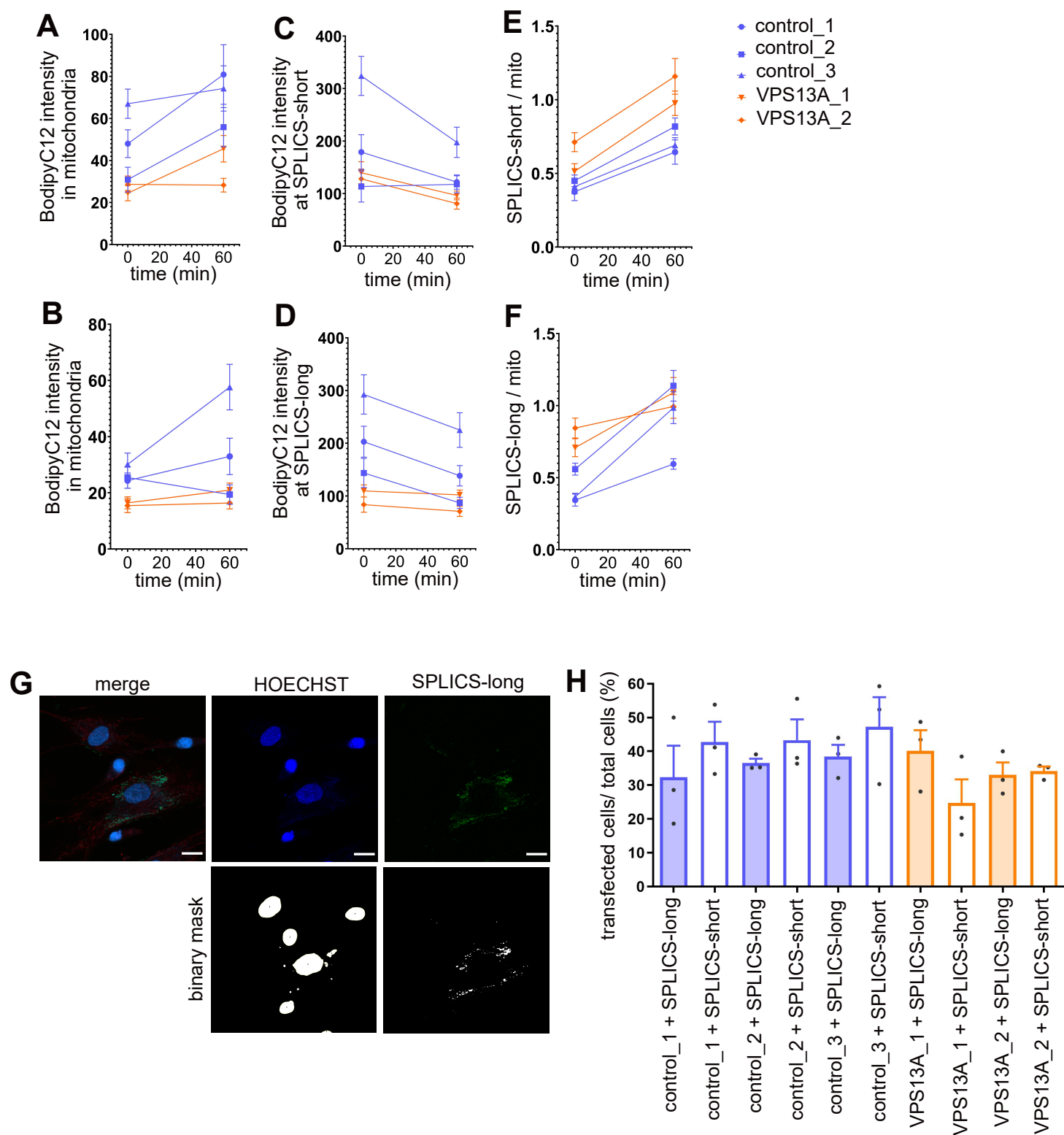

Supplement: Supplementary file 4 — Supplementary Figure S4. (A) Analysis of mean BodipyC12 intensity inside mitochondria in fibroblasts transfected with SPLICS‐short. Data from Figure 3C displayed for each individual fibroblast line (control_1: N = 3; control_2: N = 3; control_3: N = 3; VPS13A_1: N = 3; VPS13A_2: N = 3; n = 10 images per condition). (B) Analysis of mean BodipyC12 intensity inside mitochondria in fibroblasts transfected with SPLICS‐long. Data from Figure 3D displayed for each individual fibroblast line (control_1: N = 2; control_2: N = 2; control_3: N = 2; VPS13A_1: N = 2; VPS13A_2: N = 2; n = 10 images per condition). (C) Analysis of mean BodipyC12 intensity at SPLICS‐short in fibroblasts transfected with SPLICS‐short. Data from Figure 3E displayed for each individual fibroblast line (control_1: N = 3; control_2: N = 3; control_3: N = 3; VPS13A_1: N = 3; VPS13A_2: N = 3; n = 10 images per condition). (D) Analysis of mean BodipyC12 intensity at SPLICS‐long in fibroblasts transfected with SPLICS‐long. Data from Figure 3F displayed for each individual fibroblast line (control_1: N = 2; control_2: N = 2; control_3: N = 2; VPS13A_1: N = 2; VPS13A_2: N = 2; n = 10 images per condition). (E) Quantification of the SPLICS‐short area, normalized per mitochondria area in fibroblasts transfected with SPLICS‐short. Data from Figure 3G displayed for each individual fibroblast line (control_1: N = 3; control_2: N = 3; control_3: N = 3; VPS13A_1: N = 3; VPS13A_2: N = 3; n = 10 images per condition). (F) Quantification of the SPLICS‐long area, normalized per mitochondria area in fibroblasts transfected with SPLICS‐long. Data from Figure 3H displayed for each individual fibroblast line (control_1: N = 2; control_2: N = 2; control_3: N = 2; VPS13A_1: N = 2; VPS13A_2: N = 2; n = 10 images per condition). All data are mean ± SEM. (G) Fibroblasts were transfected with SPLICS‐short or SPLICS‐long (green), respectively, and 24 hours post‐transfection stained with MitoTracker Deep Red FM (red) and Hoechst (b [file MDS-41-856-s005.pdf]

**Figure S6**

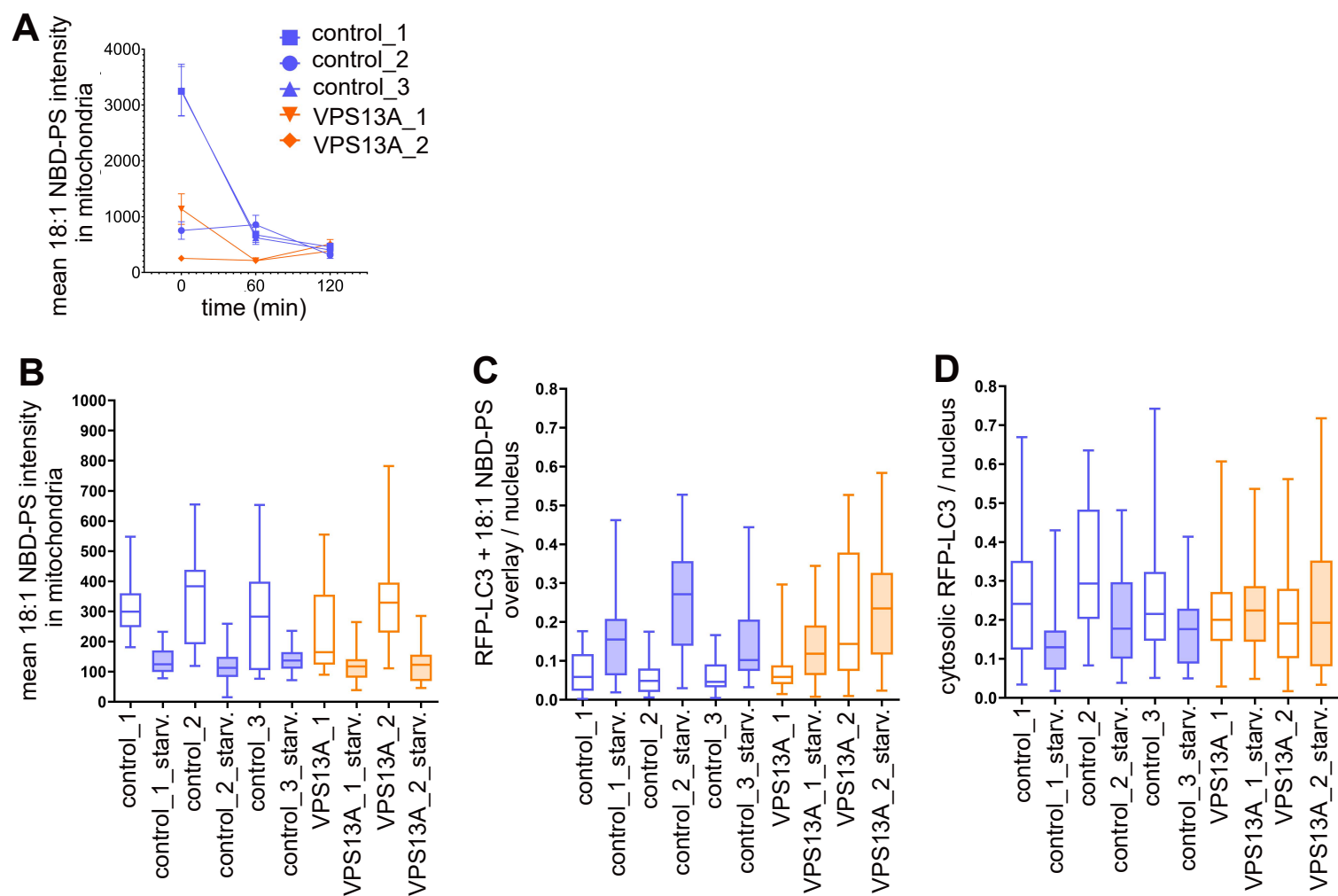

Supplement: Supplementary file 6 — Supplementary Figure S6. (A) Quantification of mean 18:1 NBD‐PS signal intensity inside mitochondria. Data from Figure 4B displayed for each individual fibroblast line (control_1: N = 3; control_2: N = 3; control_3: N = 3; VPS13A_1: N = 4; VPS13A_2: N = 4; n = 10 images per condition). All data are mean ± SEM. (B) Quantification of the mean 18:1 NBD‐PS signal inside the mitochondria. Data from Figure 4E displayed for each individual fibroblast line. (C) Quantification of colocalization events of the RFP‐LC3B and the 18:1 NBD‐PS signal, indicating autophagosomes. Data from Figure 4F displayed for each individual fibroblast line. (D) Quantification of the cytosolic RFP‐LC3B signal, outside the mitochondrial as well as outside the 18:1 NBD‐PS signal. Data from Figure 4G displayed for each individual fibroblast line (control_1: N = 3; control_2: N = 3; control_3: N = 3; VPS13A_1: N = 3; VPS13A_2: N = 4; n = 10 images per condition). All data are median ± minimum/maximum. [file MDS-41-856-s001.pdf]

Figure S7

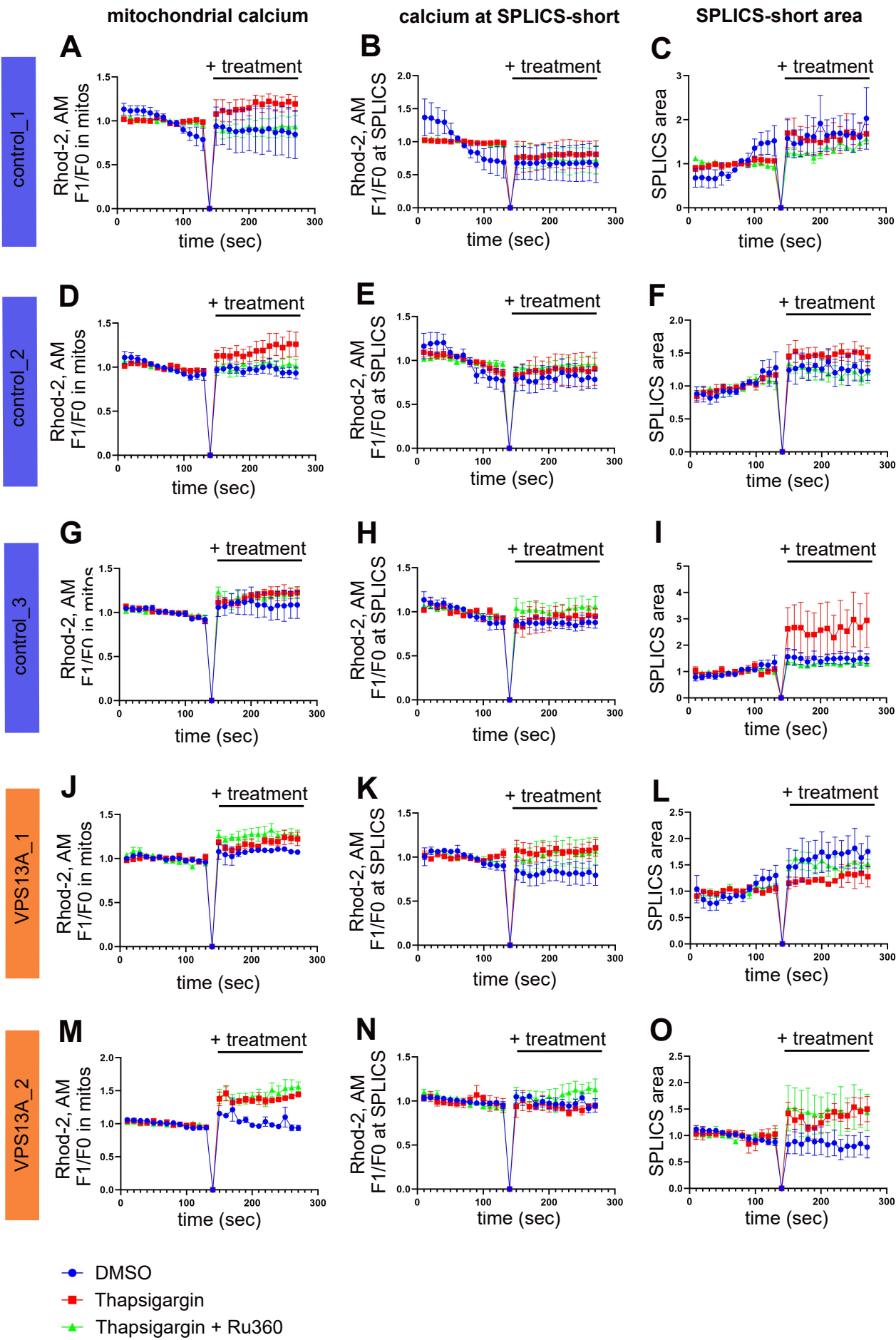

Supplement: Supplementary file 7 — Supplementary Figure S7. (A) Mean Rhod‐2, AM intensity inside mitochondria in control_1 fibroblasts (N = 3). (B) Mean Rhod‐2, AM intensity at SPLICS‐short in control_1 fibroblasts (N = 3). (C) Quantification of SPLICS‐short area in control_1 fibroblasts (N = 3). (D) Mean Rhod‐2, AM intensity inside mitochondria in control_2 fibroblasts (N = 4). (E) Mean Rhod‐2, AM intensity at SPLICS‐short in control_2 fibroblasts (N = 4). (F) Quantification of SPLICS‐short area in control_2 fibroblasts (N = 4). (G) Mean Rhod‐2, AM intensity inside mitochondria in control_3 fibroblasts (N = 4). (H) Mean Rhod‐2, AM intensity at SPLICS‐short in control_3 fibroblasts (N = 4). (I) Quantification of SPLICS‐short area in control_3 fibroblasts (N = 4). (J) Mean Rhod‐2, AM intensity inside mitochondria in VPS13A_1 fibroblasts (N = 3). (K) Mean Rhod‐2, AM intensity at SPLICS‐short in VPS13A _1 fibroblasts (N = 3). (L) Quantification of SPLICS‐short area in VPS13A _1 fibroblasts (N = 3). (M) Mean Rhod‐2, AM intensity inside mitochondria in VPS13A_2 fibroblasts (N = 3). (N) Mean Rhod‐2, AM intensity at SPLICS‐short in VPS13A _2 fibroblasts (N = 3). (O) Quantification of SPLICS‐short area in VPS13A _2 fibroblasts (N = 3). All data are mean ± SEM. [file MDS-41-856-s006.pdf]
