# Supplementary material for: VPS13A Deficiency Leads to Impaired Lipid Distribution and Alteration of Mitochondrial Calcium Homeostasis in Fibroblasts of VPS13A Disease Patients
Source: Mov Disord. 2026 Jan 19;41(4):856–69. doi: 10.1002/mds.70177 (PMC13067328; doi:10.1002/mds.70177)
Supplement: Supplementary file 5 — Supplementary Figure S5. Primary fibroblasts were investigated using high performance thin‐layer chromatography (HPTLC). Fibroblasts from three healthy controls (termed “control_1”, “control_2” and “control_3”) and two patients with VPS13A disease (“VPS13A_1” and “VPS13A_2”) were cultured as previously described in the Materials and Methods section. For each cell line, three conditions were tested: “w/o” (without starvation/without BodipyC12 (BC12) staining); “BC12” (stained with BodipyC12); and “BC12+HBSS” (stained with BodipyC12 and starved in HBSS). Fibroblasts were cultured in triplicate (three independent passages: N = 3) for all conditions and all fibroblast lines. Lipid extraction from cell pellets was performed according to the Folch method 63 , with slight modifications. After the addition of 50 μL of ice‐cold methanol (MS grade, Biosolve), containing 0.1% butylated hydroxytoluene (BHT, Sigma‐Aldrich), to the cell pellet, the solution was transferred to a new Eppendorf tube equipped with a glass insert (Knauer). The original tube was then washed with additional 50 μL of methanol, which was also transferred into the glass insert (total methanol volume: 100 μL). After the addition of 200 μL of ice‐cold chloroform (LiChrosolv®, Supelco), containing 0.1% butylated hydroxytoluene, the samples were shaken for 60 minutes at 600 rpm and 4°C using a mixing block (MB‐102, Biostep). Phase separation was achieved by adding 200 μL of water (MS grade, Biosolve) and further incubation at 600 rpm and 4°C for 10 minutes. The samples were then centrifuged at 10,000 rpm and 4°C for 10 minutes, after which the lower (organic) phase was transferred to a new Eppendorf tube containing a glass insert. The aqueous phase was then re‐extracted by adding 100 μL of chloroform to improve the extraction yield. The samples were shaken for 10 minutes at 600 rpm and 4°C, then centrifuged for 10 minutes at 10,000 rpm at 4°C. The two organic phases were then combined and dried under vacuum us [file MDS-41-856-s002.pdf]

Figure S5

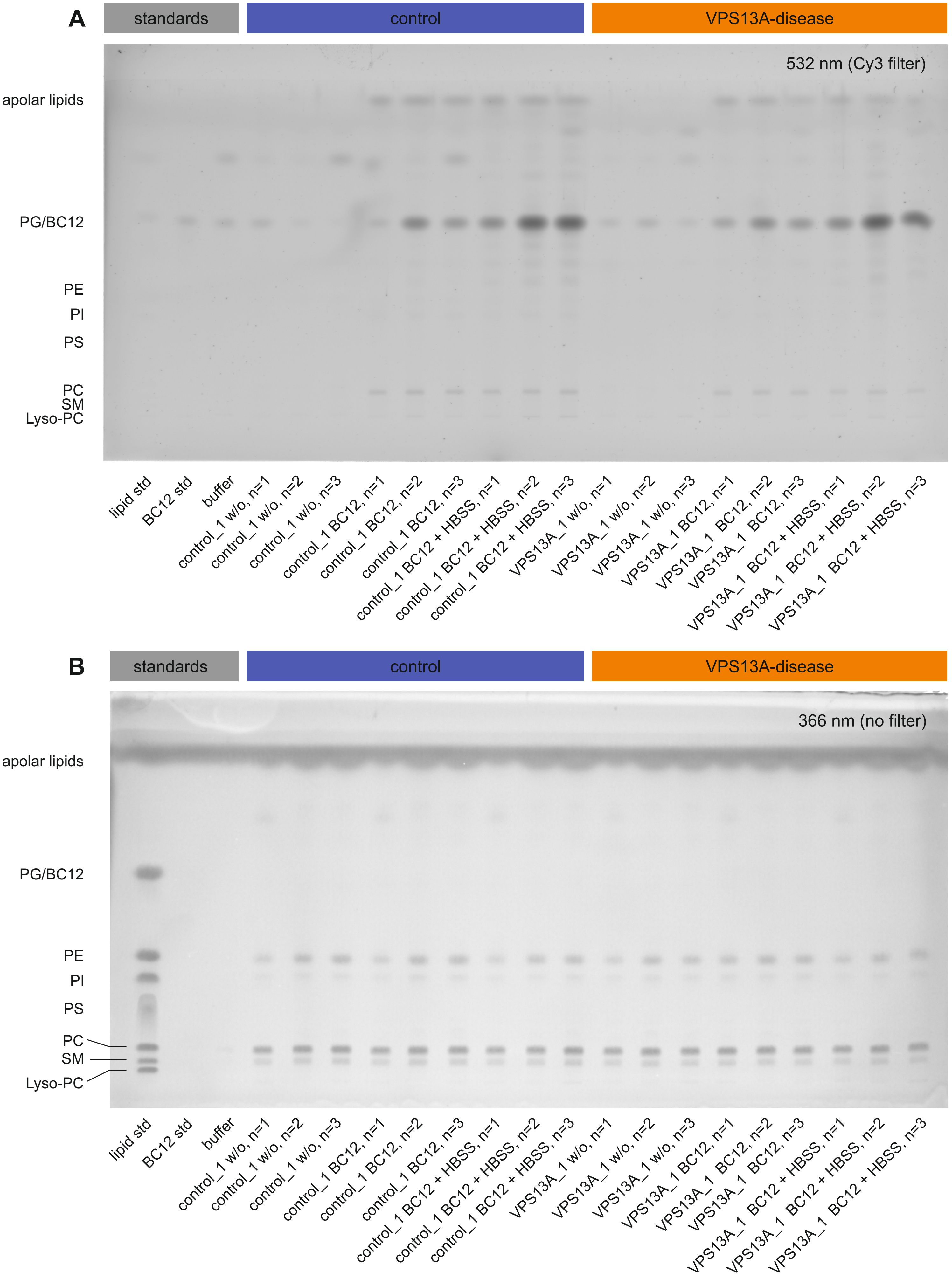
A
standards
control
VPS13A-disease
532 nm (Cy3 filter)
apolar lipids
PG/BC12
PE
PI
PS
PC
SM
Lyso-PC
lipid std
BC12 std
buffer
control_1 w/o, n=1
control_1 w/o, n=2
control_1 w/o, n=3
control_1 BC12, n=1
control_1 BC12, n=2
control_1 BC12, n=3
control_1 BC12 + HBSS, n=1
control_1 BC12 + HBSS, n=2
control_1 BC12 + HBSS, n=3
VPS13A_1 w/o, n=1
VPS13A_1 w/o, n=2
VPS13A_1 w/o, n=3
VPS13A_1 BC12, n=1
VPS13A_1 BC12, n=2
VPS13A_1 BC12, n=3
VPS13A_1 BC12 + HBSS, n=1
VPS13A_1 BC12 + HBSS, n=2
VPS13A_1 BC12 + HBSS, n=3
B
standards
control
VPS13A-disease
366 nm (no filter)
apolar lipids
PG/BC12
PE
PI
PS
PC
SM
Lyso-PC
lipid std
BC12 std
buffer
control_1 w/o, n=1
control_1 w/o, n=2
control_1 w/o, n=3
control_1 BC12, n=1
control_1 BC12, n=2
control_1 BC12, n=3
control_1 BC12 + HBSS, n=1
control_1 BC12 + HBSS, n=2
control_1 BC12 + HBSS, n=3
VPS13A_1 w/o, n=1
VPS13A_1 w/o, n=2
VPS13A_1 w/o, n=3
VPS13A_1 BC12, n=1
VPS13A_1 BC12, n=2
VPS13A_1 BC12, n=3
VPS13A_1 BC12 + HBSS, n=1
VPS13A_1 BC12 + HBSS, n=2
VPS13A_1 BC12 + HBSS, n=3

Figure S5 continued

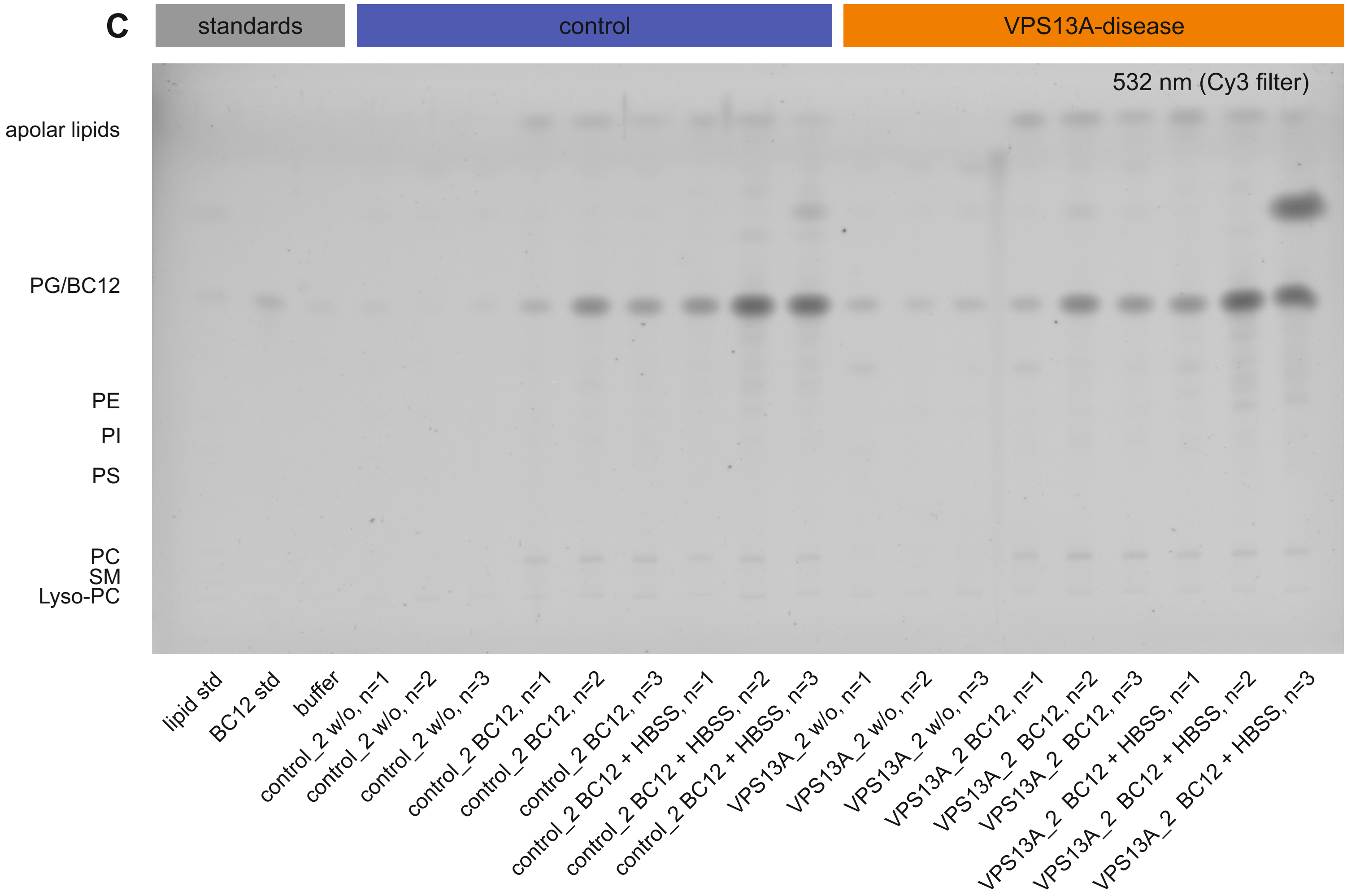

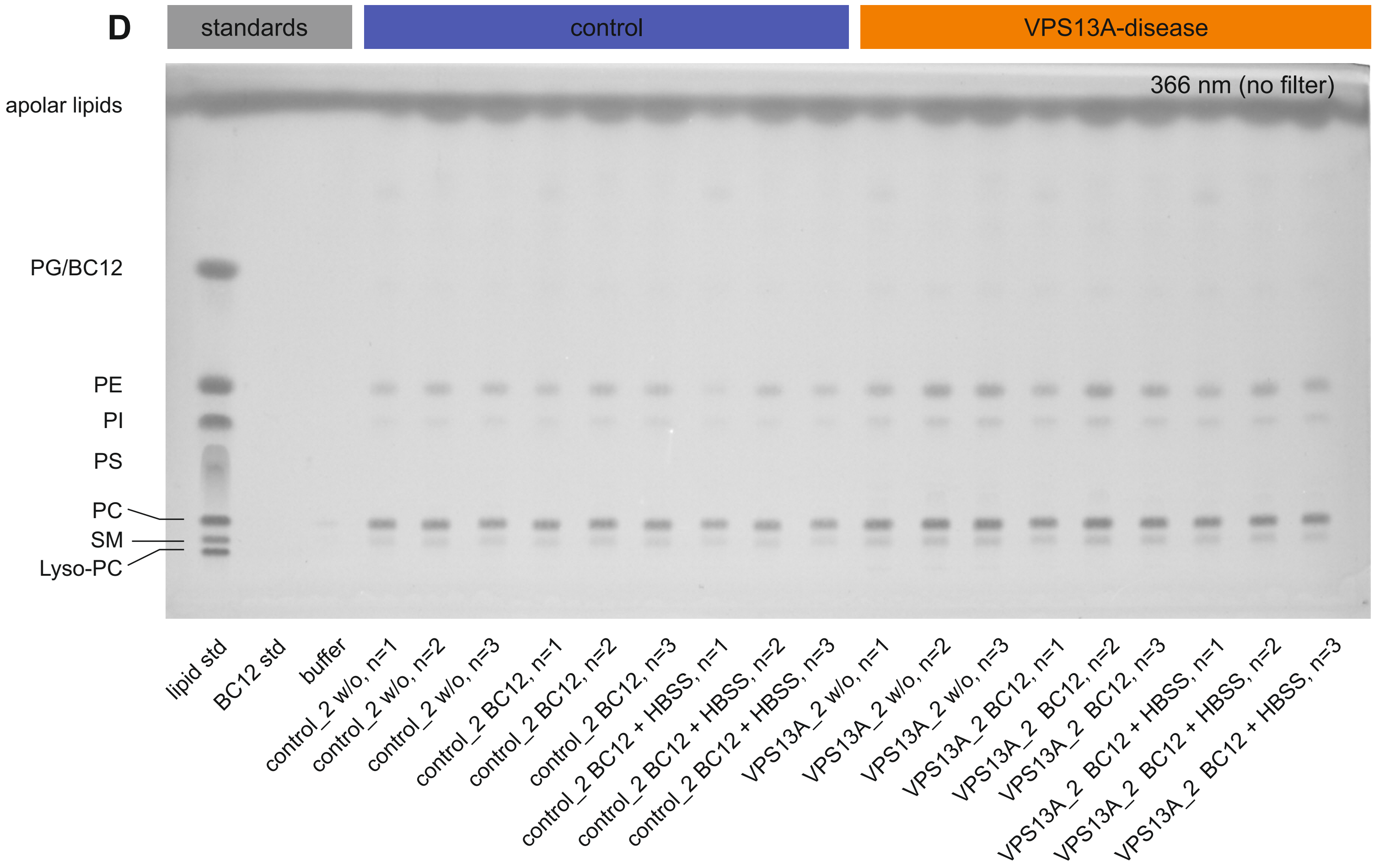

Figure S5 continued

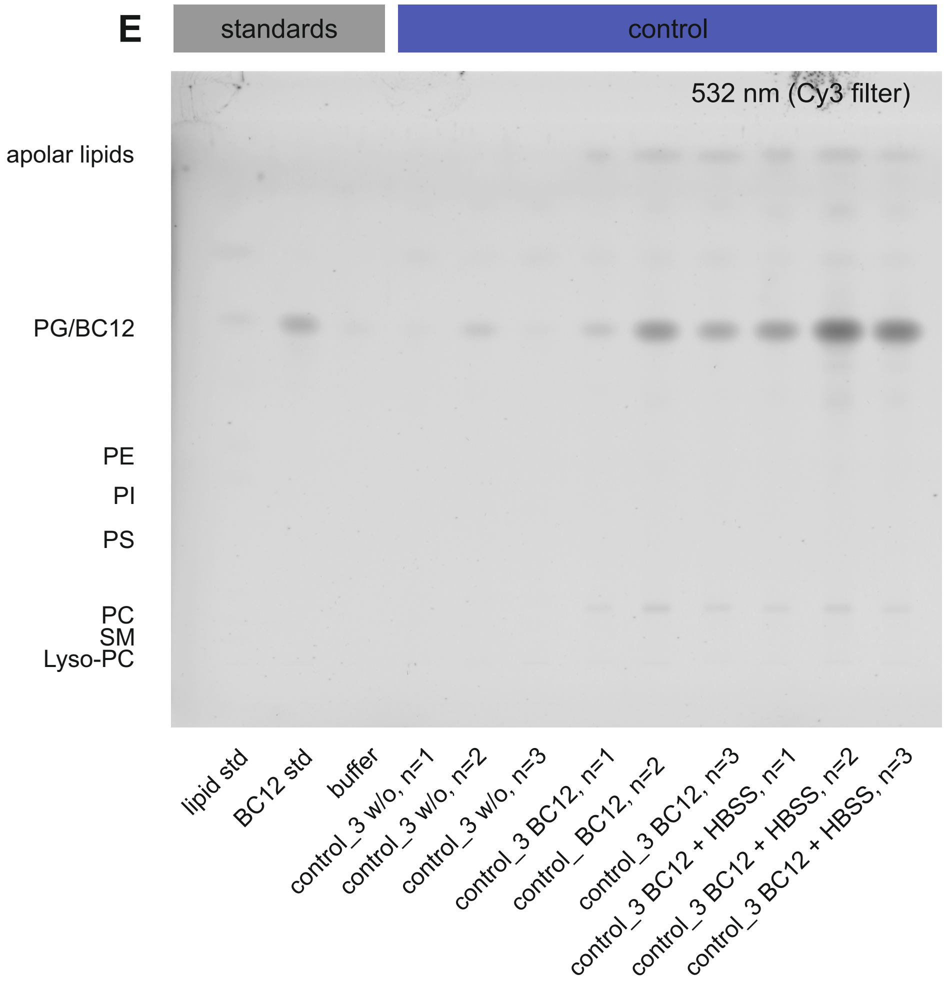

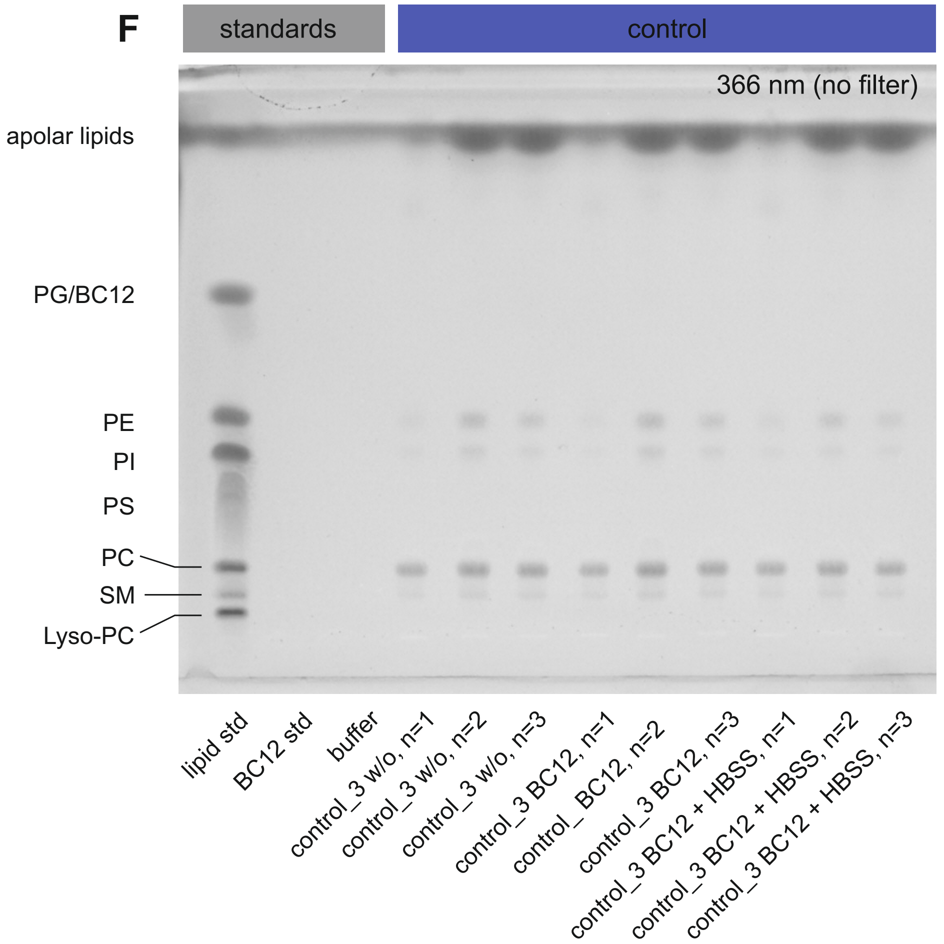

(63) FOLCH J, LEES M, SLOANE STANLEY GH. A simple method for the isolation and purification of total lipides from animal tissues. J Biol Chem. 1957;226(1):497–509.

(64) Fuchs B, Schiller J, Süss R, Schürenberg M, Suckau D. A direct and simple method of coupling matrix-assisted laser desorption and ionization time-of-flight mass spectrometry (MALDI-TOF MS) to thin-layer chromatography (TLC) for the analysis of phospholipids from egg yolk. Anal Bioanal Chem. 2007;389(3):827–34. doi:10.1007/s00216-007-1488-4
